# Quantitative metagenomics reveals unique gut microbiome biomarkers in ankylosing spondylitis

**DOI:** 10.1186/s13059-017-1271-6

**Published:** 2017-07-27

**Authors:** Chengping Wen, Zhijun Zheng, Tiejuan Shao, Lin Liu, Zhijun Xie, Emmanuelle Le Chatelier, Zhixing He, Wendi Zhong, Yongsheng Fan, Linshuang Zhang, Haichang Li, Chunyan Wu, Changfeng Hu, Qian Xu, Jia Zhou, Shunfeng Cai, Dawei Wang, Yun Huang, Maxime Breban, Nan Qin, Stanislav Dusko Ehrlich

**Affiliations:** 1 Institute of Basic Research in Clinical Medicine, College of Basic Medical Science, Zhejiang Chinese Medical University, Hangzhou, 310053 China; 2 Realbio Genomics Institute, Shanghai, 200123 China; 3 State Key Laboratory for Diagnosis and Treatment of Infectious Diseases, Department of Infectious Diseases, the First Affiliated College of Medicine, Zhejiang University, Hangzhou, 310003 China; 4 Collaborative Innovation Center for Diagnosis and Treatment of Infectious Diseases, Zhejiang University, Hangzhou, 310003 China; 5 INRA, Institut National de la Recherche Agronomique, Metagenopolis, Jouy en Josas, 78350 France; 6 Rheumatology Division, Ambroise-Paré Hospital, AP-HP, 9, avenue Charles-de-Gaulle, 92100 Boulogne-Billancourt, France; 7 King’s College London, Centre for Host-Microbiome Interactions, Dental Institute Central Office, Guy’s Hospital, London Bridge, London, SE1 9RT UK

**Keywords:** Ankylosing spondylitis, Human gut microbiome, Biomarkers, Pathogenesis

## Abstract

**Background:**

The assessment and characterization of the gut microbiome has become a focus of research in the area of human autoimmune diseases. Ankylosing spondylitis is an inflammatory autoimmune disease and evidence showed that ankylosing spondylitis may be a microbiome-driven disease.

**Results:**

To investigate the relationship between the gut microbiome and ankylosing spondylitis, a quantitative metagenomics study based on deep shotgun sequencing was performed, using gut microbial DNA from 211 Chinese individuals. A total of 23,709 genes and 12 metagenomic species were shown to be differentially abundant between ankylosing spondylitis patients and healthy controls. Patients were characterized by a form of gut microbial dysbiosis that is more prominent than previously reported cases with inflammatory bowel disease. Specifically, the ankylosing spondylitis patients demonstrated increases in the abundance of *Prevotella melaninogenica*, *Prevotella copri*, and *Prevotella* sp. C561 and decreases in *Bacteroides* spp. It is noteworthy that the *Bifidobacterium* genus, which is commonly used in probiotics, accumulated in the ankylosing spondylitis patients. Diagnostic algorithms were established using a subset of these gut microbial biomarkers.

**Conclusions:**

Alterations of the gut microbiome are associated with development of ankylosing spondylitis. Our data suggest biomarkers identified in this study might participate in the pathogenesis or development process of ankylosing spondylitis, providing new leads for the development of new diagnostic tools and potential treatments.

**Electronic supplementary material:**

The online version of this article (doi:10.1186/s13059-017-1271-6) contains supplementary material, which is available to authorized users.

## Background

Ankylosing spondylitis (AS) is a systemic, chronic, inflammatory autoimmune disease characterized by the inflammation of the axial skeleton, the peripheral joints, and the attachments of ligaments and entheses. Prevalence of AS is 0.2–0.54% in the ethnic Han Chinese population [1] and approximately 0.5% in the USA [2]. AS mainly affects the physical function, quality of life, and the working ability of young men and consequently imposes a considerable burden on both the patients and society [3]. The delay between the onset of symptoms and diagnosis is up to 8–10 years due to the insidious progression of AS. The most effective current medication, tumor necrosis factor (TNF) blockade, does not seem to work in all patients to arrest bone erosion or syndesmophyte formation. Considering the diagnostic delay and insufficient therapeutic options, a better understanding of the pathogenesis of this disease is necessary.

AS is regarded as a genetic disease and is strongly associated with HLA-B27 [4]. To date, other HLA variants and non-MHC loci have been identified as the genetic biomarker of AS through genome-wide association studies [5, 6]. A growing body of evidence indicated AS and inflammatory bowel disease (IBD) shared similarity of genetic risk factors and etiopathogenesis [7, 8]. The interaction between host genetics and gut microbiome may be involved in IBD pathogenesis [9]. Hence, AS may also be a microbiome-driven disease. The most notable association is the acknowledged ability of *Chlamydia trachomatis* and *Klebsiella* to trigger HLA-B27-associated disease; molecular mimicry has been suggested as a pathophysiological mechanism [10]. However, these conclusions are mainly based on serological tests and have not been universally accepted. Interestingly, a distinct AS gut microbial signature was reported in a comparison of patients with healthy controls on the basis of 16S ribosomal RNA gene sequencing. In these patients, the abundance of *Lachnospiraceae*, *Veillonellaceae*, *Prevotellaceae*, *Porphyromonadaceae*, and *Bacteroidaceae* was significantly increased, while that of *Ruminococcaceae* and *Rikenellaceae* was notably decreased [11]. In contrast to Crohn’s disease, the microbial biomass did not differ between the AS patients and controls. How these alterations in the microbial community structure correlate with the homeostasis of the host, however, is obscure.

During the last decade, novel sequencing technologies have revolutionized the field of microbiology and the role of the microbiome in inflammatory and autoimmune diseases has recently gained great attention. Significant differences were reported for the microbiomes of patients with autoimmune diseases such as Crohn’s disease [12], ulcerative colitis [13, 14], rheumatoid arthritis (RA) [15], systemic lupus erythematous (SLE) [16, 17], and psoriasis [18]. A quantitative metagenomics analysis developed by the MetaHIT consortium revealed a significant loss of gut microbial richness associated with the risk of metabolic syndrome [19] and co-morbidities associated with liver cirrhosis (LC) [20]. No study has yet used this approach to analyze AS.

In this study, we conducted a quantitative metagenomics study in 211 Chinese individuals. These participants were divided into a discovery cohort of 73 AS patients and 83 healthy controls as well as a validation cohort of 24 patients and 31 healthy controls.

## Results

### Updated integrated gene catalog

The first integrated gene catalog (IGC, including type 2 diabetes [T2D], IBD, human microbiome project [HMP], and MetaHIT individuals) contains 9,879,896 genes from 1267 gut metagenomes [21]. To better assess the relationship between AS and the gut microbiome, we first assembled the genes from the AS patients and our healthy controls (patient characteristics are reported in Additional file 1: Table S1) and then constructed an updated integrated gene catalog, denoted as IGC2 hereafter, which encompasses the IGC, LC, and AS gene catalogs. The gene catalog for the AS gut microbiome contained 2,319,710 non-redundant open reading frames (ORFs), whereas the LC gut microbial catalog contained 2,688,468 genes [20]. Together they contained 517,488 genes not present in the IGC1. We added them to the IGC1 and obtained a new catalog named IGC2 (Table 1 and Additional file 2: Figure S1a) that was used in the remainder of the study.

**Table 1:** The statistics of gene catalogs

Gene catalog	Year of publication	Sample numbers (#)	Gene numbers (#)	Total bases (bp)	Average length (bp)
MetaHIT	2010	124	3,299,822	2,323,171,095	704
T2D (+MetaHIT)	2012	145 (+124)	4,267,985	3,081,440,484	722
LC	2014	181	2,688,468	2,017,496,337	750
AS (+LC_H^*^)	This time	73 (+83)	2,319,710	1,682,594,586	725
IGC	2014	1267	9,879,896	7,436,156,055	753
IGC2	This time	1521	10,397,384	7,766,094,066	747

*LC_H* healthy samples in LC project

### Phylogenetic differences between AS patients and healthy controls

**Fig. 1:**
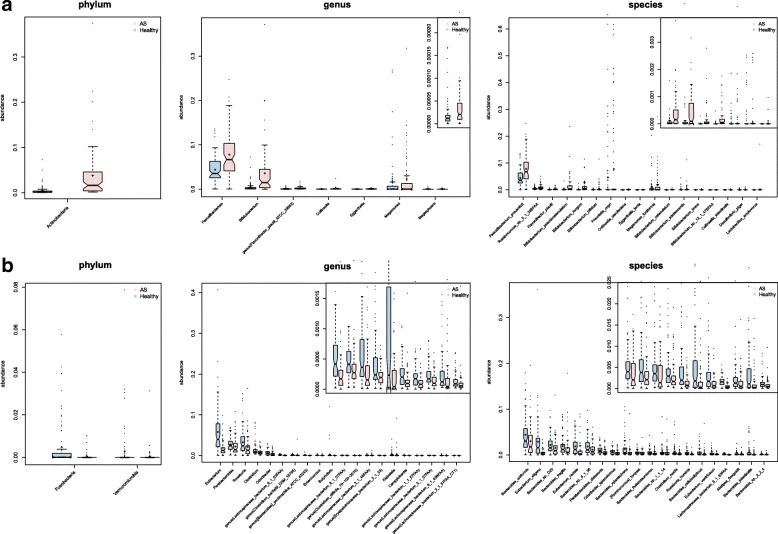
Differences of phylogenetic abundance between AS patients and healthy controls. The phylotypes that were increased (**a**) or decreased (**b**) in the AS patients at the phylum, genus, and species levels. *Red* and *blue* indicate the AS patients and healthy controls, respectively. The phylogenetic abundance of phyla that had mean values less than 1% and that of genera and species that were less than 0.01% were excluded. After exclusion, Wilcoxon rank-sum tests were applied to identify the differentially abundant phyla, genera, and species. Among these, the highest medians of the phylogenetic abundance in the enriched cohort were drawn as *boxplots*

The sequencing reads (Additional file 1: Table S2) were aligned against 8743 reference genomes from the NCBI and HMP (Additional file 1: Table S3). The diversity of the gut microbiomes for the AS patients and the healthy controls was similar at genus level (Additional file 2: Figure S1a) but was significantly higher in the controls at species level (Additional file 2: Figure S1b). This result indicated that the genera were represented by more species in the healthy participants. Analysis of phylotypes with a median relative abundance larger than 1% and 0.1% at the genus and species levels, respectively, indicated that Bacteroidetes, Firmicutes, Proteobacteria, and Actinobacteria were the four dominant taxa in both the AS patients and healthy controls, which, in agreement with other studies [20], represented > 99% of the microbiome data. However, in contrast to the results of Costello et al. [22], at the phylum level there were no significant differences between the two groups in the abundance of Bacteroidetes or the ratio of Bacteroides to Firmicutes (Additional file 2: Figures S2, S3), but the abundance of Actinobacteria was significantly greater (*p* = 1.50e-15, Wilcoxon rank-sum test) in the AS patients (Fig. 1a) and that of Fusobacteria (*p* = 9.02e-08, Wilcoxon rank-sum test) and Verrucomicrobia (*p* = 2.86e-04, Wilcoxon rank-sum test) was lower (Fig. 1b). Consistent with this result, an increase in the Actinobacteria was also observed at the genus level, as four of the top five enriched genera (*Neisseria*, *Bifidobacterium*, *Collinsella*, *Rothia*, and *Actinomyces*; Additional file 1: Table S4) belonged to Actinobacteria. Concomitantly, the AS microbiome was depleted of gram-negative bacteria: *Enterobacter* (*p* = 3.89e-10, Wilcoxon rank-sum test); and *Citrobacter* (*p* = 5.54e-07, Wilcoxon rank-sum test). The genera belonging to Enterobacteriaceae were enriched in the healthy controls, as were *Fusobacterium* and a genus most closely related to *Lachnospiraceae* bacterium (Additional file 1: Table S4).

The most abundant species in both the AS and healthy control groups were primarily from the *Bacteroides* genus. Of the 20 species for which the abundance was most decreased in the AS group, ten were *Bacteroides* spp. (Fig. 1b). It has been reported that *Bacteroides* spp. are reduced in RA [23] and IBD [24]. However, in other diseases, such as psoriasis and celiac disease, Bacteroides spp. were found to increase. These observations suggest that phylogenetically related species can be differentially enriched in different diseases. Noticeably, of the species that increased the most in the AS patients, 13 were Actinobacteria (Fig. 1a), specifically from the genus of *Bifidobacterium* (*p* = 2.42e-12, Wilcoxon rank-sum test), some of which are often used as probiotics. However, it has been reported that *B. bifidum* (*p* = 1.96e-08, Wilcoxon rank-sum test), *B. longum* (*p* = 1.38e-12, Wilcoxon rank-sum test), and *B. pseudocatenulatum* (*p* = 9.69e-07, Wilcoxon rank-sum test) can induce a TH2-driven immune response [25] and the glycopolymers of *B. bifidum* may play a role in the pathogenesis of autoimmune thyroid diseases through the mechanism of molecular mimicry [26, 27].

Furthermore, compared with healthy controls, the microbial communities in the AS cases were characterized by a higher abundance of Prevotellaceae including *Prevotella melaninogenica* (*p* = 5.91e-09, Wilcoxon rank-sum test), *Prevotella copri* (*p* = 1.18e-03, Wilcoxon rank-sum test), and *Prevotella* sp. C561 (*p* = 1.12e-07, Wilcoxon rank-sum test). This observation is consistent with the data of Costello [22]. The study by Lin [28] noted marked effects of HLA (human leukocyte antigen)-B27 on the gut microbiota; and an increase in the *Prevotella* spp. was observed in HLA-B27 transgenic rats. *P. copri* may stimulate an immune reaction that then targets joint tissues and this species was strongly correlated with the disease severity in untreated new-onset RA patients [23] whereas *P. melaninogenica* can interact with human lactoferrin.

### Gene markers and functional differences between AS patients and healthy controls

The AS patients exhibited a reduced richness of the gut microbiome compared with the healthy controls (414,289 ± 129,035 and 539,071 ± 189,228, respectively, *p* = 6.68e-06, Wilcoxon rank-sum test) and the overall distribution of their gene counts was clearly shifted toward lower values (Additional file 2: Figure S4a, b). The Shannon-Wiener diversity index (*p* = 1.50e-07, Wilcoxon rank-sum test) and the Simpson diversity index (*p* = 3.57e-06, Wilcoxon rank-sum test) also reflected the lower gut microbiome richness of the AS patients (Additional file 2: Figure S4).

To identify the differentially abundant genes, Wilcoxon rank-sum test was applied to 73 AS patients and 83 controls. Among these participants, 23,709 genes were identified: 6238 were more abundant in AS patients and 17,471 in the healthy controls, using the threshold value of > 1e-7 for mean relative abundance and < 1e-4 for q (Additional file 2: Figure S5a). Compared with those for IBD and LC, the degree of gut dysbiosis in AS was intermediate, IBD being lowest and LC highest, as deduced from the *p* value distributions (Additional file 2: Figure S5b).

To analyze the functional difference between the patients’ and controls’ microbiomes, we used the KEGG (Kyoto Encyclopedia of Genes and Genomes) annotation, which was available for 40% (3,684,628/10,397,384) of all the ICG2 genes. The most abundant KEGG orthologs in both groups were those associated with carbohydrate metabolism (Additional file 2: Figure S6). The most enriched orthologs in the AS patients were related to membrane transport, similar to findings for LC [20] and T2D [29]. In contrast, the most prevalent markers among the AS patients included those involved in cell motility, membrane transport, metabolism of cofactors and vitamins, and signal transduction (Additional file 2: Figures S7, S8). Furthermore, the microbiota regulates the intestinal immune responses primarily through the production of microbe-associated molecular patterns (MAMPs) such as lipopolysaccharides (LPS) and flagellin.

Moreover, the module related to proteasome functions was more abundant in the AS samples whereas those for glycosaminoglycan metabolism, secondary metabolites biosynthesis, and symbiosis were elevated in healthy controls (Additional file 2: Figure S9). The proteasome is a multi-subunit proteolytic complex that is involved in the degradation of many cytosolic and nuclear proteins that regulate pathways critical for cell survival. This complex is widely expressed in eukaryotic cells and some prokaryotes such as the Archaea and Actinobacteria. The enrichment of this module was consistent with the higher abundance of Actinobacteria in the AS patients. Furthermore, all of the proteasome-associated genes that were identified as being differentially abundant in this study belonged to the bacterial proteasome (Additional file 1: Table S8).

### Metagenomic species (MGS) in AS disease and comparison with other diseases

**Fig. 2:**
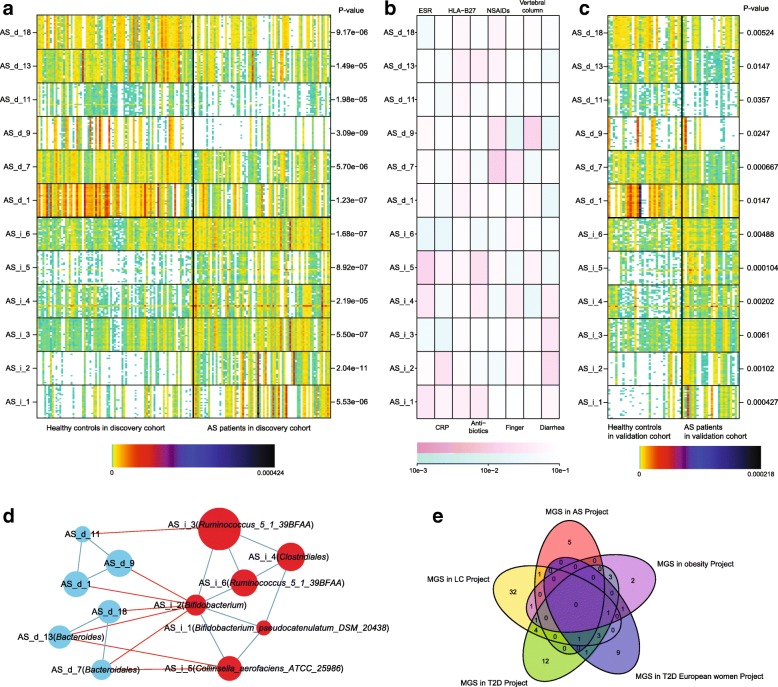
MGSs in AS patients and healthy controls and association with clinical indices. **a** The abundance of 12 MGSs are shown as *heatmaps*: the discovery set (n = 156) is on the *left* and the validation set (n = 55) is on the *right*. The *colors* denote the variation in abundance (*white* indicates zero; *black* indicates the highest abundance). **b** The association between MGSs and clinical index is shown in the *middle*: the darker the color, the greater the intensity. *Violet* indicates a positive correlation with each index (the index partition in first row), *green* indicates a negative correlation with each index (the index partition in first row). The 25 genes in each MGS for which the mean abundance values were the highest are shown in the *heatmap*. **c** On the right of the *heatmap*, the Wilcoxon rank-sum test *p* values for the mean abundance of the 25 “marker genes” are indicated. Above the heatmap, the *color key* shows how the color variation indicates the abundance. **d** The networks of the 12 MGSs reflect the interaction between them. The notes represent the MGSs and the note size is proportional to the mean abundance of the genes in the MGS. The *red lines* represent the negative correlation between the two notes and the *blue lines* represent the positive correlation between the two notes. **e** The *Venn diagram* of the MGS in AS Project, LC Project, T2D Project, T2D European Women Project, and Obesity Project (Additional file 1: Table S11)

MGS is a gene group in which all of the genes are deferentially abundant between patients and healthy controls, the genes in the same MGS have consistent abundance among individuals. We grouped the genes into clusters denoted MGSs [30] according to the gene abundance (Fig. 2). Of the 6238 genes enriched in the AS cohort, 2594 genes were clustered into six MGSs, whereas of the 17,471 genes enriched in the healthy controls, 5291 were clustered in 23 MGSs (Additional file 1: Table S9). All of the MGS were significantly different in the discovery cohort and 12 were also significant in the validation cohort (all six of the AS-enriched and six of the control-enriched). Four of the MGSs that were enriched in the AS patients could be annotated to the strain level; two of them are *Bifidobacterium* and *B. pseudocatenulatum_*DSM_20438, which is consistent with the phylogenetic analysis that identified an increase in the Bifidobacteria as presented in a previous section.

We compared the MGS from different studies, targeting the AS study, LC [20], and obesity [19]; we included the T2D [29] and European Women T2D studies [31], although it is known that these two studies are confounded to a certain degree by the metformin treatment administered to some of the patients [31]. We found that only a few species were common to these different diseases (Additional file 1: Table S11). *Clostridium bolteae*, *Clostridium symbiosum*, and *Clostridium ramosum* were enriched in patients in the T2D Project and the Obesity Project, which showed that some species in some genus were similar in obesity patients and T2D patients. Importantly, a majority of the MGS markers were disease-specific (Fig. 2e), indicating that they could be a foundation for novel non-traumatic monitoring and classification approaches based on human gut microbiome for different chronic diseases.

### Unknown taxonomic organism identified by population metagenomics technology

To further explore the bacterial species associated with AS that was not captured by the gene-based approach presented above, we grouped the AS-specific catalog genes (1,708,140 genes that were with abundance in at least ten samples from 2,319,710 genes) into clusters of ≥ 700 genes, denoted “big CAGs (Co-abundance gene groups).” This was facilitated by using the approach described by Nielsen et al. [32] across the 211 individuals of our cohort (AS patients and healthy controls). We obtained 199 big CAGs and validated them in the validation cohort (Additional file 2: Figure S10). Of these, 63 (31.7%) could be assigned taxonomically (Additional file 1: Table S12, Additional file 2: Figure S11), consistent with previous data that indicated that most gut bacterial species have no closely related reference genomes [32]. In a similar manner, we obtained and validated 755 clusters of 100–699 genes, hereafter denoted “small CAGs” [32]: 583 (77.2%) of these 755 were unknown taxonomic organisms.

To identify the clusters (included the big CAGs and small CAGs) associated with AS, the low abundance clusters were filtered (Online Methods) and the Wilcoxon rank-sum test was used (false discovery rate [FDR] < 0.01). There were 62 clusters, which included 16 big CAGs and 46 small CAGs (Additional file 1: Table S12). Among these, 25 cluster markers (six big CAGs and 19 small CAGs) were confirmed in the validation cohort (*p* < 0.05). Compared with the 12 clusters of differentially abundant genes shown in Fig. 2, MGS (AS_i_1) belongs to cag4343, MGS (AS_i_2) belongs to cag521, MGS (AS_i_3) belongs to cag1907, the majority of the MGS (AS_d_1) belongs to cag555, and the others contain some genes that overlapped with the big CAGs and small CAGs. Of the 62 clusters, 16 clusters were directly annotated to the strain level. The other clusters should also be strain-level clusters, but their strain names were unknown (Additional file 1: Table S12). Among these, ten clusters were annotated to the species level, eight clusters were annotated to the genus level, four clusters were annotated to the order level, and 24 clusters were completely unknown. All of these 62 clusters and the 12 clusters of differentially abundant genes shown in Fig. 2 were used as cluster markers to construct a new classification model for AS based on the human gut microbiome.

### New classification model for AS

**Fig. 3:**
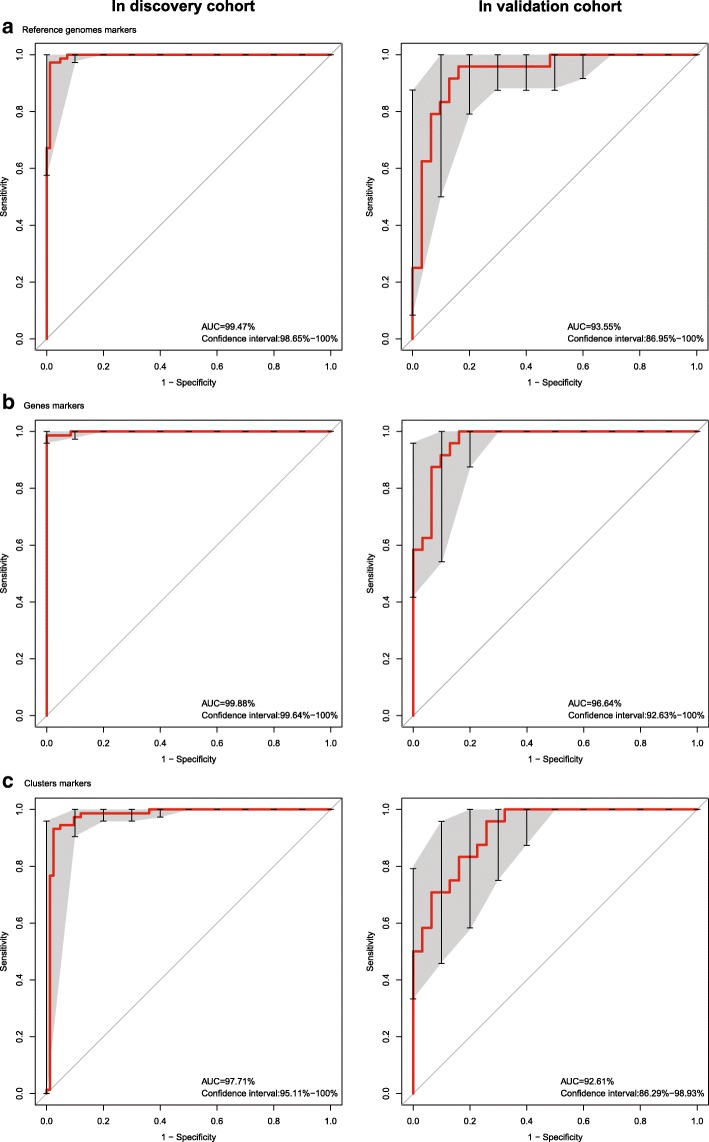
Receiver-operating characteristic (ROC) curves of the sequenced reference genome markers, gene markers, and cluster markers. **a** Classifier based on 25 sequenced reference genome markers and the ROC curves for the discovery and validation cohorts. **b** Classifier based on 35 gene markers and the ROC curves for the discovery and validation cohorts. **c** Classifier based on 62 cluster markers and the ROC curves for the discovery and validation cohorts. The discovery cohort was the 156 samples that were used to identify the markers; the validation cohort was the 55 samples that were used to validate the markers such as those shown in Fig. 2

We constructed classification algorithms (classifiers) to identify AS patients using three types of biomarkers: sequenced reference genomes, genes, and clusters, which included the MGSs and CAGs. With the Matthews Correlation Coefficient (MCC) optimization selection and the Support Vector Machine (SVM), three classifiers were constructed (Online Methods) based on the three types of bio-markers (marker details in Additional file 1: Table S13), and the receiver-operating characteristic (ROC) curves were drawn (Fig. 3). During the classifier construction, all 210 differentially abundant sequenced reference genome markers (Additional file 1: Table S14) were input and 33 sequenced reference genome markers were picked after MCC optimization selection (Additional file 1: Table S13). The top 100 differentially abundant genes with smallest *p* values (<1.1e-12) were input and finally 30 genes were used. All 62 of the differentially abundant clusters (including metagenomic species and CAGs) and 12 MGSs were merged together and a final group of 62 cluster markers (11 MGSs, 14 big CAGs, and 37 small CAGs) were used (Additional file 1: Table S13).

From the ROC curves (Fig. 3), we found that the gene markers (area under ROC curve [AUC]) = 96.64% in the validation cohort) were better than the sequenced reference genome markers (AUC = 93.55% in the validation cohort) because the gene markers were not limited to known taxonomic organisms. The cluster markers were high-level bio-markers based on gene markers, although they were not good enough (AUC = 92.61% in the validation cohort) because they contained many unknown taxonomic organisms. The cluster markers and gene markers were important complements to the sequenced reference genome markers.

All three of the classifiers that were based on human gut microbiome could be used as new classification models for AS.

## Discussion

Here, we integrated the AS gene catalog with the IGC and LC gene catalogs to generate reference genes. Approximately 17% additional genes (232,446 genes) could be aligned effectively with the expanded catalog as opposed to the AS gene catalog alone. The new catalog should facilitate quantitative characterization of the metagenomic, metatranscriptomic, metaproteomic, and metabolomic data from the gut microbiome to better understand their variations across population groups and cross-talk between the microbiota and its host. Due to few AS participants who did not take any medicine, a major limitation of our study is that we do not know whether medication use is driving the difference seen in AS patients compared with healthy controls. This will require further study, such as the analysis of newly diagnosed AS patients.

**Fig. 4:**
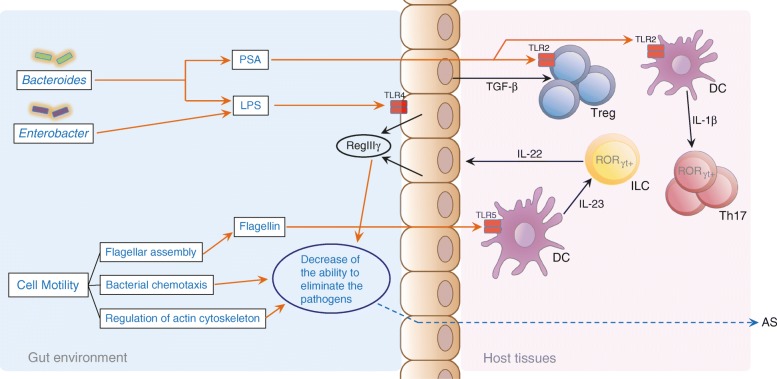
A *schematic diagram* of the main functions of the gut microbes associated with AS. The *red text* denotes enriched in the AS patients; the *blue text* denotes depleted in the AS patients; the *orange lines* and *arrows* denote the actions initiated by the gut microbes or functional in the gut environment in this study; the *black line* and *arrows* denote the known actions and mechanisms functional in the host tissues as previously reported; the *blue dashed line* and *arrows* denote the inferred actions and mechanisms in the host tissues that were associated with the gut microbes. Here, we present some information regarding the influence of the gut microbiota on both the innate and adaptive immune responses. With respect to the innate immune responses (see Fig. 1), RegIIIγ hyposecretion caused by decreased levels of LPS and flagellin and accompanied by depletion of bacterial chemotaxis, regulation of the actin cytoskeleton, Fc gamma R-mediated phagocytosis, and NOD-like receptor signaling result in the dysbiosis of the gut microbiome and the onset of AS. With respect to the adaptive immune responses (see Fig. 2), a reduction in the levels of Polysaccharide A (PSA), which is mainly produced by the Bacteroides, may directly or indirectly influence the differentiation of the Treg cells and thereby contribute to AS

It has recently become clear that the influence of the microbiota extends beyond the intestinal tract and affects the systemic immune system. In the current study, we present compelling evidence that the gut microbiome is altered in AS. The alteration might have a role in the pathogenesis of AS, possibly by modulating both the innate and adaptive immune systems. The recognition of MAMPs by the intestinal epithelial cells induces secretion of the antimicrobial peptide RegIIIγ, which mediates colonization resistance in the gut [33]. A decrease in the content of LPS caused by the depletion of gram-negative bacteria and an associated reduction in the flagellar assembly could lead to RegIIIγ hyposecretion, which could promote the dysbiosis of the gut microbiome that is associated with AS (Fig. 4, Additional file 2: Figure S7).

The epithelium of the human intestinal tract has necessarily evolved mechanisms to prevent or limit the activation of cellular immune-inflammatory stress responses and the transcription factor NF-κB is often involved in those immune and inflammatory responses. It has been reported that NF-κB is activated through the tightly regulated phosphorylation, ubiquitination, and proteolysis of the inhibitor molecule, IκB. As previously mentioned, proteasomes are found in eukaryotes, archaea, and actinobacteria, and the central component of all proteasomes, the core particle, is similar in overall structure [34]. Actinobacteria have been shown to modify proteins by the attachment of a small protein modifier termed prokaryotic ubiquitin-like protein, which can target proteins for degradation by proteasomes [34–36]. It has been reported that multiple species of non-pathogenic bacteria can attenuate the NF-κB pathway via specific inhibition of IκB-α ubiquitination whereas the ubiquitin-mediated degradation of IκB-α is mediated by a common Ub ligase, E3-SCF^β-TrCP^. With the information above, and our results demonstrating that *Actinobacteria* been enriched in AS patients, we speculated that *Actinobacteria* might modulate the ubiquitination of IκB-α. This in turn would allow the activation of NF-κB signaling and the accumulation of proinflammatory factors in patients with AS. This could facilitate the development of AS. A large study showed that proteasomes have an obvious correlation with autoimmune diseases including SLE, RA, scleroderma, and others, and the serum proteasome concentration in many autoimmune diseases patients was significantly increased [37]. Furthermore, a hypothesis has been proposed that the ubiquitin/proteasome system, autophagy, or cross-talk among different proteolytic pathways may possibly contribute to the pathogenesis of T1D, another polygenic autoimmune disease that occurs in individuals who are genetically predisposed on the basis of their human leukocyte antigen types. The ubiquitin-proteasome system has been used as a clinical target in the treatment of multiple myeloma and an exploration of the potential use of proteasome inhibition for autoimmune diseases including AS is pending. The elucidation of a possible functional role for the increase in the gut bacterial proteasome components in AS would require further studies.

## Conclusions

The human gut mirobiome of AS patients was clearly different from that of healthy controls. There was loss of richness of the gut microbiome in AS patients. Our results demonstrated that some alteration of gut microbiome is associated with developments of AS, evidenced by the changes in genes, pathways, and various taxonomic levels. According to previous reports, we inferred that some biomarkers participate in the pathogenesis or the development process of AS, such as *Bifidobacterium*, *Prevotella melaninogenica*, *Prevotella copri*, *Prevotella* sp. C561, and the gene markers related to bacterial proteasome. Other markers that were not clear in pathogenesis could provide new information for further research. The classification model based on biomarkers in the gut microbiome might provide a new direction for future clinical examination and diagnosis. Lastly, discovery of the associated microbes of AS in the gut microbiome may help us to seek more treatments for this disease.

## Methods

### Patient information

After informed consent was provided, a total of 211 individuals (97 patients with AS and 114 healthy controls) were enrolled in this study from three hospitals (Zhejiang Provincial Hospital of Traditional Chinese Medicine, the Second Affiliated Hospital of Zhejiang Chinese Medical University, and Zhejiang Province People Hospital). The 97 individuals (57 men, 40 women) aged 14–71 years with AS were diagnosed on the basis of the modified New York criteria for AS [38]. The clinical information of the participants (gender, age, BMI, clinical manifestation, blood HLA-B27 level, erythrocyte sedimentation rate [ESR], C-reactive protein [CRP], alanine aminotransferase [ALT], aspartate transaminase [AST], albumin [ALB], globulin [GLB], urea nitrogen [BUN], and creatinine [Cr]) was collected, and the Bath Ankylosing Spondylitis Functional Index (BASFI) [39] and Bath Ankylosing Spondylitis Disease Activity Index (BASDAI) [40] were calculated. Among them, age, BMI, disease duration, and non-steroidal anti-inflammatory drug treatment showed no effects in this research (Additional file 2: Figure S12a–d). The information of healthy controls was collected in the First Affiliated College of Medicine, Zhejiang University. The healthy controls (72 men, 42 women) were aged 23–70 years and were free from any history of IBD or any rheumatic disease. AS patients and controls with gastrointestinal tract disorders and those undergoing treatment with antibiotics within one month prior to the stool collection were excluded. Patients with severe systemic diseases or hepatitis were excluded.

A dietary questionnaire that recorded the complete diet information and dietary habits was completed before the blood sample collection. This questionnaire was used to exclude individuals that had specific dietary habits such as alcohol consumption or a completely vegetable-based diet. The clinical information on the participants is presented in Additional file 1: Table S1. The clinical diagnosis and blood examination data for all individuals were obtained from the hospitals.

### AS patient fecal sample collection

All of the fresh fecal samples from the AS patients were transferred immediately from the hospital to the laboratory [20] and divided into ten aliquots of 200 mg. All samples were stored at –80 °C until DNA extraction.

### Using the public data of healthy controls

The data of healthy controls were published before and could be downloaded from European Bioinformatics Institute European Nucleotide Archive (ERP005860). The average clean data of them was 3.84 Gbp. Because the AS patients in this study kept a similar daily diet (Southeast China dietary habit: rice as staple food and bland dish styles) with these healthy controls, the data of healthy controls were used. In order to make the two groups (AS patients and healthy controls) as comparable as possible, all the experimental protocols and bioinformatics pipelines for each sample in this study were in line with the published paper [20], including DNA extraction, library construction, sequencing, quality control, host genome filtering, and other bioinformatics analysis methods. Non-metric multi-dimensional scaling (NMDS) analysis for all the samples (AS patients and healthy controls) based on phylogenetic abundance profiling showed that the samples of the AS patients and healthy controls were randomly distributed in the NMDS space (Additional file 2: Figure S12a–d); it showed that the batch effects in this study were minor.

### DNA extraction and library construction

The DNA was extracted from each frozen fecal sample (200 mg) using the phenol/trichloromethane DNA extraction method. The quality of DNA was measured using a NanoDrop instrument (Thermo Scientific, used to estimate the DNA concentrations) and agarose gel electrophoresis (used to measure the molecular sizes). TruSeq DNA HT Sample Prep Kit was used for library construction. The quality of the DNA library was estimated using Qubit to estimate the DNA concentration and Agilent 2100 (used to measure the insert sizes).

### Sequencing

All the samples were sequenced in the Illumina Hiseq 2000 in BGI-Shenzhen and PE100 sequencing strategy was used; each sequencing run had 9–11 samples and the sequencing depth of each sample must be at least 3 Gbp. The insert sizes of all samples were in the range of 275–450 bp.

### Quality control and host genome filtering

The raw reads from the AS patients and healthy controls that had 50% low quality bases (quality ≤ 20) or more than five ambiguous bases were excluded. Subsequently, reads with low quality tails (quality ≤ 20) were trimmed, the remaining reads were mapped to the human genome (hg19) by SOAP alignment (v2.21) [41], and the matching reads were removed as being contaminants from the host genome.

### Assembly and repeated assembly

All high-quality reads from the AS discovery cases were assembled by SOAP de novo (v2.04) [42], which has been effectively used in metagenomics research. We randomly chose ten samples to test the performance of various k-mer sizes in the range of 21–59 in increments of 2 in the assembly; the conclusion was that the k-mers that led to the highest N50 varied from 51 to 59. Therefore, five k-mers 51, 53, 55, 57, and 59 were used in the assembly and the N50-highest k-mer was chosen. After the scaffold was obtained, we split the scaffold into “scaftigs” by removing the ambiguous bases and discarded the scaftigs whose length were less than 500 bp. The publicly available results of the assembled healthy controls were used directly for the scaffolds and processed by the same method. The original clean reads were mapped to the scaffolds by SOAP alignment and the unused reads were pooled and assembled again. The first repeated assembly was within 20 sets (Additional file 1: Table S5), while the repeated assembly was within four sets (Additional file 1: Table S6). The k-mers in the repeated assembly were all set to 55.

### Gene prediction and gene catalog construction

The coding sequences were predicted from the split scaffolds from both the original assembly and the repeated assembly by MetaGeneMark (v2.8) [43], which used a hidden Markov model to predict the ORFs (Additional file 1: Table S7). After filtering the genes whose lengths were less than 100 bp, the remaining sequences were clustered by CD-hit [44] to construct a non-redundant gene catalog. Two genes whose identity and coverage were greater than 0.95 and 0.9, respectively, were merged together and the longer one was regarded as the representative sequence. After that, this gene catalog was merged with the IGC and LC gene catalogs using CD-hit with the same parameters.

### Phylogenetic abundance profiling and gene abundance profiling

The clean reads were mapped to the reference genomes collected from NCBI and HMP using the SOAP aligner and the phenotype profiling was evaluated using the number of hits for the reads against a certain reference genome. For certain species, reads that were paired-matched to related genomes were split into two parts: (1) U: reads match this genome only; and (2) M: reads also match another genome, and the abundance of the species was also split into two parts, Ab(U) and Ab(M). The unique part Ab(U) was calculated as the number of reads divided by the length of the genome. For the multiple part Ab(M), each of the reads in set M was assigned to several parts according to the unique abundance of species with which the reads matched [20]. The formulae were as follows:

$$ \begin{array}{c} Ab\left(\mathrm{S}\right)= Ab(U)+ Ab(M)\\ {} Ab(U)={\displaystyle \sum_{i=1}^U1/l}\\ {} Ab(M)={\displaystyle \sum_{i=1}^M\left(Co*1\right)/l}\\ {}Co=\frac{Ab(U)}{{\displaystyle \sum_{i=1}^N Ab\left({U}_i\right)}}\end{array} $$

where Ab(U) and Ab(M) indicate the unique part and the multiple part of the species abundance, respectively, and *l* indicates the length of genome.

On the basis that the genes are usually shorter than the genome, when calculating gene abundance, it is too strict to choose only paired-match reads. We relaxed the limits such that the following reads were also pooled into calculation: the read was single-end matched to a reference, but the other end of the read was beyond the range of genes (the insert size was considered to be 800 bp). The remain steps were identical to those use for the calculation of the abundance of the species.

### Gene counting and alpha diversity

The number of genes that were detected in each sample was counted. To eliminate the effect of the various data sizes among the samples, all reads were randomly sampled to 9 M (million) reads and the same method was applied to calculate the abundance and count the number of genes. The Shannon-Weiner alpha diversity and the Simpson alpha diversity were calculated and compared between the AS patients and healthy controls (Additional file 2: Figure S4).

### Differential gene identification

On the basis that genes with too low an abundance in both AS patient samples and healthy control samples might not appropriately reflect the actual situations, the genes with median abundance less than 1e-7 in the AS patient group or the healthy control group were discarded. The differential genes were identified by the Wilcoxon rank-sum test with a threshold FDR < 0.001. The estimated pi_0_ values (ratio of the null hypothesis in Additional file 2: Fig. S5a) were calculated using the q value R package [45].

### Gene functional analysis

The protein sequences of the genes in the merged gene catalog were aligned to the KEGG protein database [41] using BLAT [46]. After filtering the match scores under 60, the genes were assigned to the KEGG orthology groups on the basis of the highest scores of the matches. The abundance of a certain KO (KEGG orthology group) was calculated as the sum of the abundances of genes that were assigned to this group. To identify the KOs that were associated with AS, the Wilcoxon rank-sum test was performed with FDR < 0.005 and the observed differential KEGG orthology groups were assigned to the KEGG pathways.

### MGS identification

For the identified differentially expressed genes, hierarchical clustering was applied using Spearman’s correlation coefficient for the abundance of the genes with a clustering threshold of 0.9. After abandoning clusters with less than 25 genes, a second hierarchical clustering was performed using Spearman’s correlation coefficient for the mean abundance of genes in each cluster with a new threshold was 0.8. The final clusters of genes were called MGSs [20].

### CAG identification

To assess the abundance of all genes that were detected in more than ten samples, a canopy algorithm was applied [47]. The T1 threshold for the canopy algorithm was a Pearson correlation coefficient > 0.95 and a Spearman’s correlation coefficient > 0.7, whereas the T2 threshold was a Pearson correlation coefficient > 0.9. After the first clustering process and exclusion of canopies that contained only one gene, according to mean abundance of the clusters, a canopy-like algorithm was applied. For a group that contained canopies obtained by first clustering, a new canopy will merge into this group if the Pearson correlation coefficient between this canopy and more than 70% of the canopies in this group > 0.9. After the former process, the second clusters may have overlaps. Thus, for a certain gene that is in more than one cluster, the distances between the gene and the clusters to which it belongs were calculated and the closest cluster was chosen. The new clusters were called CAGs and those that contained more than 700 genes were selected for further research [32]. After filtering the median abundance lower than 1E-8 in both groups, the Wilcoxon rank-sum tests were applied to select the CAG markers that had FDRs < 0.0005.

### Phylogenetic annotation of MGSs/CAGs

To annotate the MGSs/CAGs into a taxonomy, all of the genes of each MGS/CAG were mapped to the NCBI databases for bacteria, fungi, and viruses using BLAT. The strict matching results were regarded as valid matches if the identity > 0.95 and coverage > 0.9. If more than 90% genes were annotated into only one certain taxonomy, the MGS/CAG was annotated into this phenotype.

### Self-learning classifier

We choose three types of data, the abundance of reference genomes markers, gene markers, and clusters markers in the discovery set as the features with which to build the classifiers. The redundant features were filtered: several subsets of all features were chosen by the mRMR algorithm (the side Channel Attack R package) [48] and the leave-one-out cross-validation LDA (Linear Discriminant Analysis) (the paleoMAS R package) was applied. The ones for which the highest MCC were obtained were chosen to build a SVM classifier (the e1071 R package). The number of features in each subset were reduced from the original number by step 5 (one in the case of the MGS). The ROC figures for both the discovery set and validation set were drawn using the pROC R package.

### Data access

The sequencing data of the 97 AS samples have been submitted to NCBI Sequence Read Archive under accession number SRP100575.

## Additional files

Additional file 1: **Table S1.** Phenotype information of AS patient individuals and health controls in discovery stage (156 samples) and validation stage (55 samples). **Table S2.** Data production and quality control of 156 samples in discovery stage and 55 samples in validation stage. **Table S3.** The 8743 reference genomes from NCBI and HMP (downloaded on 15 Dec 2013). **Table S4.** The differentially abundant genus in AS patients (n = 73) and healthy controls (n = 83). **Table S5.** Assembly result of 156 samples in discovery stage. **Table S6.** The improvement with the repeatedly assembly. **Table S7.** Gene prediction of 156 samples in discovery stage. **Table S8.** Genes with abundance which belong to proteasome modules. All the differentially abundant genes identified in this study only belong to bacterial proteasome. **Table S9.** The taxonomic annotation of MGSs. **Table S10.** The phenotypic correlation analysis (*p* value) of 12 MGSs according to different clinical groups. **Table S11.** Comparison of the MGS in different diseases. **Table S12.** The taxonomic annotation of CAGs (Gene number ≥ 100). **Table S13.** The details of the best markers selected for five monitoring and classification models based on five kinds of bio-markers. **Table S14.** The 210 differentially abundant sequenced reference genome markers used for classification training. (XLSX 870 kb)

Additional file 2: **Figure S1a.** *Venn diagram* of three existing human gut gene catalogs. **Figure S1b.** Diversity of genera and species between AS patients and healthy controls. **Figure S2.** The Bacteroidetes/Firmicutes ratio in the AS patient group and in the healthy control group. **Figure S3.** Phylogenetic abundance under phylum, genus, and species levels between AS patients and healthy controls. **Figure S4.** Loss of richness of the gut microbiome in AS. **Figure S5.** The distribution of *p* values. **Figure S6.** The distribution of KEGG functional categories (statistics in Level 2) for all genes and differentially abundant genes. **Figure S7.** The distribution of detail pathways in four KEGG functional categories which were quite different between AS-enriched genes and control-enriched genes in Figure S6. **Figure S8.** The distribution of eggNOG functional categories for AS related markers. **Figure S9.** The distribution of KEGG module categories for AS related markers shown by number and percentage. **Figure S10.** *Heatmap* of the abundance of a random metagenomic species in both sequencing data and downloaded data. **Figure S11.** Taxonomic annotation of genes in CAGs by NT database. **Figure S12.** The NMDS (non-metric multidimensional scaling) analysis based on phylogenetic abundance profiling of all the 156 samples in the discovery cohort. (DOCX 4671 kb)

## Abbreviations

AS: Ankylosing spondylitis
AUC: Area under ROC curve
CAGs: Co-abundance groups
HLA: Human leukocyte antigen
HMP: Human microbiome project
IBD: Inflammatory bowel disease
IGC: Integrated gene catalog
KEGG: Kyoto Encyclopedia of Genes and Genomes
KO: KEGG orthology group
LC: Liver cirrhosis
LPS: Lipopolysaccharides
MAMPs: Microbe-associated molecular patterns
MCC: Matthews correlation coefficient
MGS: Metagenomic species
ORFs: Open reading frames
ROC: Receiver-operating characteristic
SVM: Support vector machine
T1D: Type 1 diabetes
T2D: Type 2 diabetes
TNF: Tumor necrosis factor.
