# Correction to: Quantitative metagenomics reveals unique gut microbiome biomarkers in ankylosing spondylitis

**DOI:** 10.1186/s13059-017-1352-6

**Published:** 2017-11-08

**Authors:** Chengping Wen, Zhijun Zheng, Tiejuan Shao, Lin Liu, Zhijun Xie, Emmanuelle Le Chatelier, Zhixing He, Wendi Zhong, Yongsheng Fan, Linshuang Zhang, Haichang Li, Chunyan Wu, Changfeng Hu, Qian Xu, Jia Zhou, Shunfeng Cai, Dawei Wang, Yun Huang, Maxime Breban, Nan Qin, Stanislav Dusko Ehrlich

**Affiliations:** 1 Institute of Basic Research in Clinical Medicine, College of Basic Medical Science, Zhejiang Chinese Medical University, Hangzhou, 310053 China; 2 Realbio Genomics Institute, Shanghai, 200123 China; 3 State Key Laboratory for Diagnosis and Treatment of Infectious Diseases, Department of Infectious Diseases, the First Affiliated College of Medicine, Zhejiang University, Hangzhou, 310003 China; 4 Collaborative Innovation Center for Diagnosis and Treatment of Infectious Diseases, Zhejiang University, Hangzhou, 310003 China; 5 INRA, Institut National de la Recherche Agronomique, Metagenopolis, Jouy en Josas, 78350 France; 6 Rheumatology Division, Ambroise-Paré Hospital, AP-HP, 9, avenue Charles-de-Gaulle, 92100 Boulogne-Billancourt, France; 7 King’s College London, Centre for Host-Microbiome Interactions, Dental Institute Central Office, Guy’s Hospital, London Bridge, London, SE1 9RT UK

## Correction

Upon publication of the original article [1], it was noted that references 11 and 22 were miscited in the reference list. Both references should be cited as:

Costello ME, Ciccia F, Willner D, Warrington N, Robinson PC, Gardiner B, Marshall M, Kenna TJ, Triolo G, Brown MA: Brief Report: Intestinal Dysbiosis in Ankylosing Spondylitis. Arthritis Rheumatol 2015, 67:686-691
