# Supplementary material for: Quantitative metagenomics reveals unique gut microbiome biomarkers in ankylosing spondylitis
Source: Genome Biol. 2017 Jul 27;18:142. doi: 10.1186/s13059-017-1271-6 (PMC5530561; doi:10.1186/s13059-017-1271-6)

Additional files 1: Supplementary figures for:

**Quantitative metagenomics reveals unique gut microbiome biomarkers in ankylosing spondylitis**

**Supplementary Figures**

**Figure S1. a. Venn diagram of three existing human gut gene catalogs.** IGC2 is composed of three human gut gene catalogs, AS gene catalog, LC gene catalog and the integrated gene catalog (IGC). Shared parts and specific parts are indicated. **b. Diversity of genera and species between AS patients and healthy controls.** Diversity of genera was similar between AS patients and healthy controls, while diversity of species in healthy controls was higher than that in AS patients.


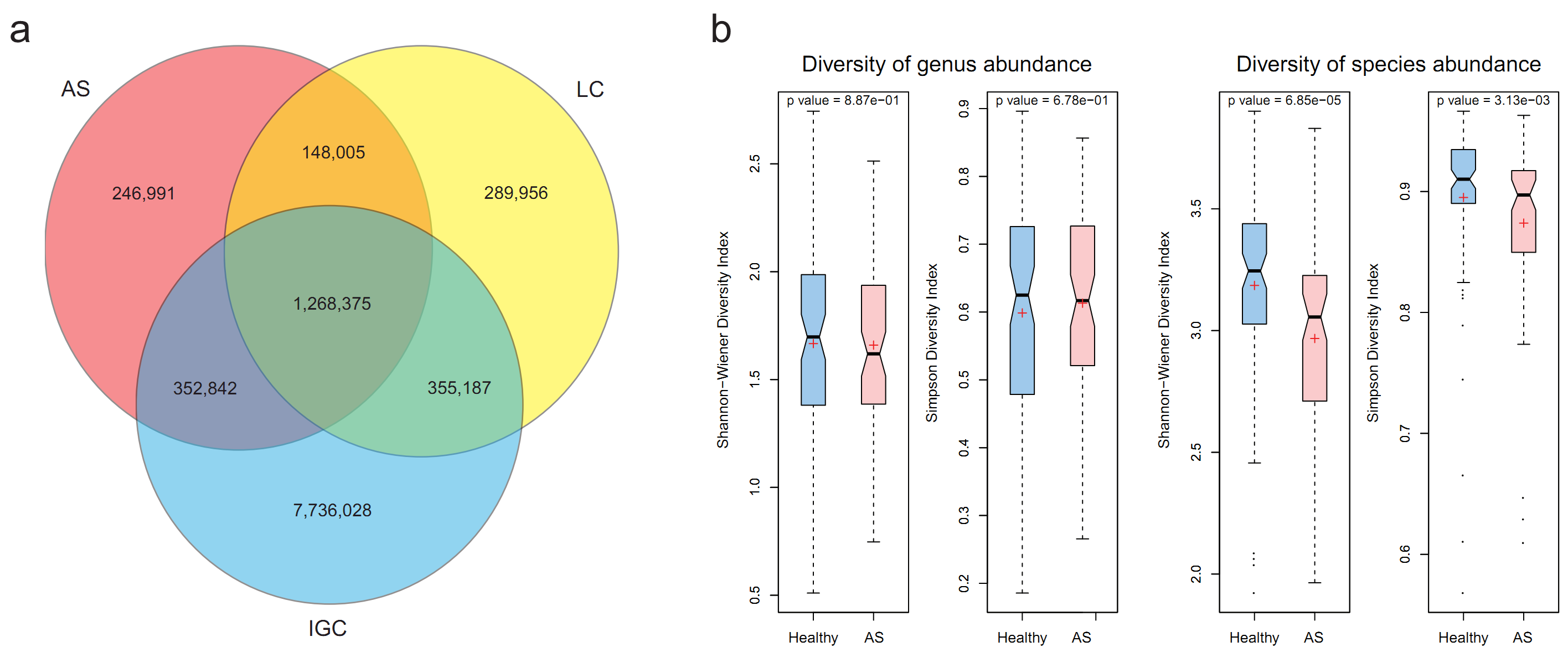


**Figure S2. The Bacteroidetes/Firmicutes ratio in AS patient group and in healthy control group**. The majority of Bacteroidetes/Firmicutes ratio in AS patients and healthy controls are similar, the p value (p=0.853) shows there is no obvious difference between them.


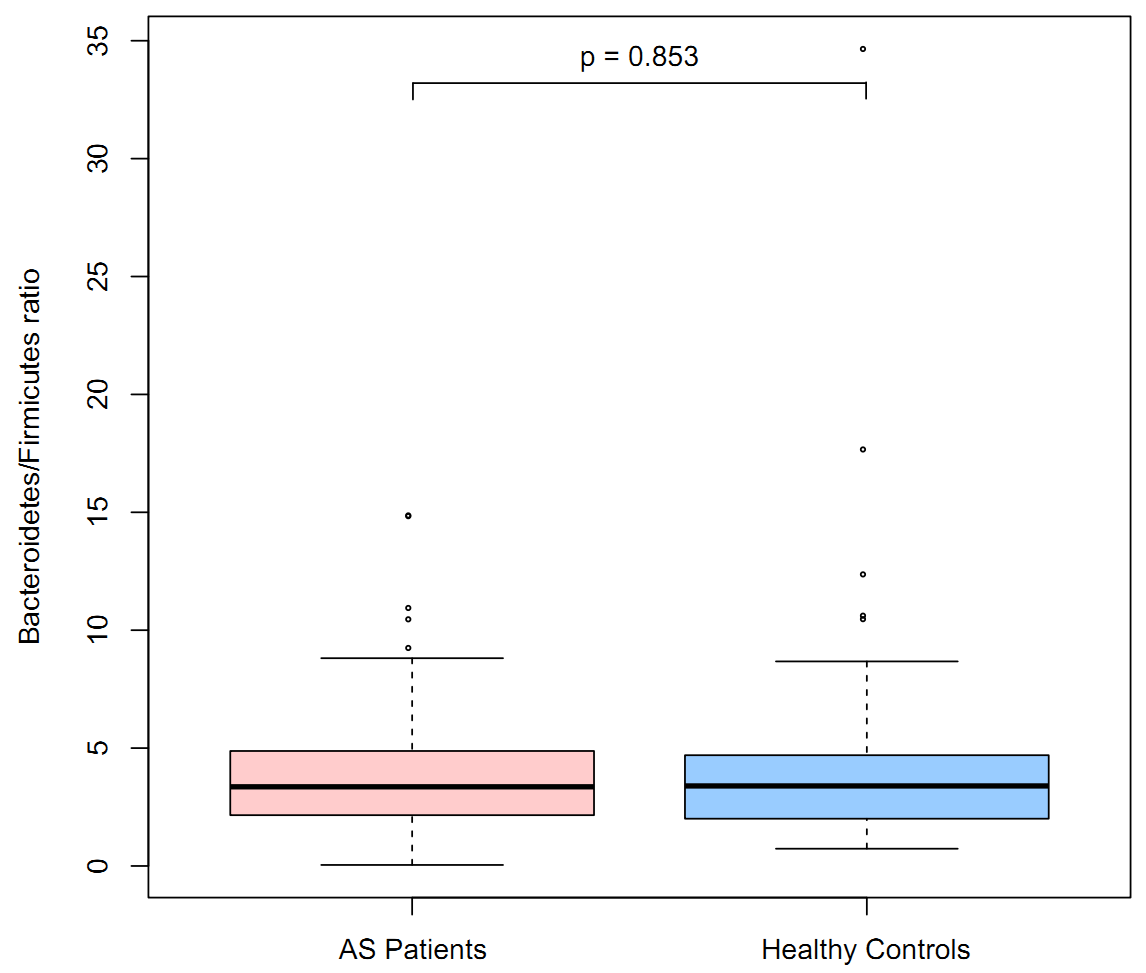


**Figure S3. Phylogenetic abundance under phylum, genus, species levels between AS patients and healthy controls.**

**a**, Phylogenetic abundance of phyla and the 30 most abundant genera and species in ankylosing spondylitis patients are shown. **b**, Phylogenetic abundance of phyla and the 30 most abundant genera and species in healthy controls are shown. The boxes represent the interquartile range (IQR), and the lines in boxes indicate the median values. The whiskers show the lowest and highest values within 1.5 interquartile range (IQR) from the first and third quartiles. The points denote the outliers. The plus signals represent the mean values of abundance. The color of box indicates the phylum of each species. Red, green, blue, cyan and magenta represent Bacteroidetes, Firmicutes, Proteobacteria, Actinobacteria and other phyla, respectively.


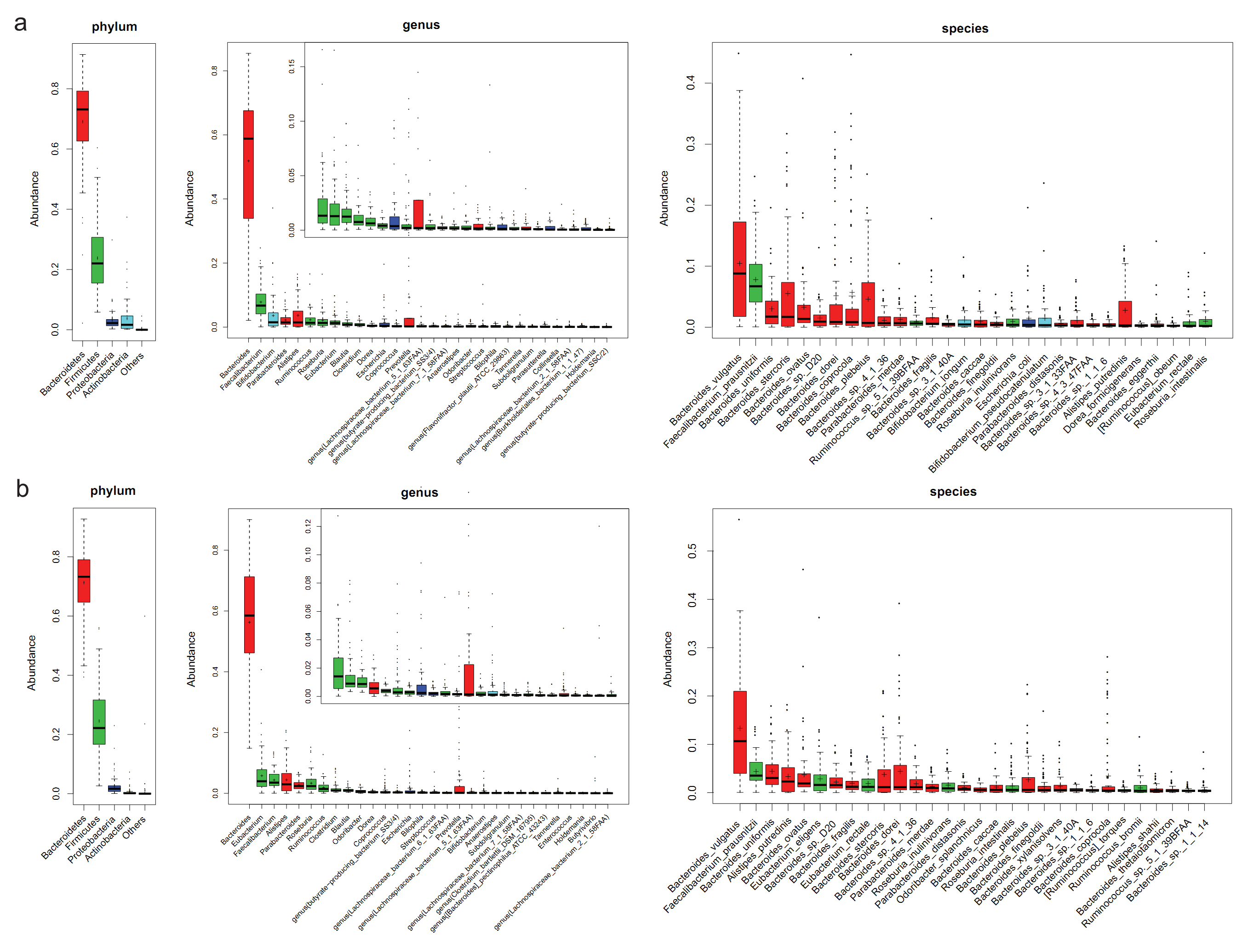


**Figure S4. Loss of richness of the gut microbiome in AS**

**a.** All reads were used to compute the numbers of genes (left, inset shows the box-plot), the Shannon-Weiner and Simpson diversity index (right). **b.** Reads were downsized to 9M reads per sample, to avoid the effect of sequencing depth.


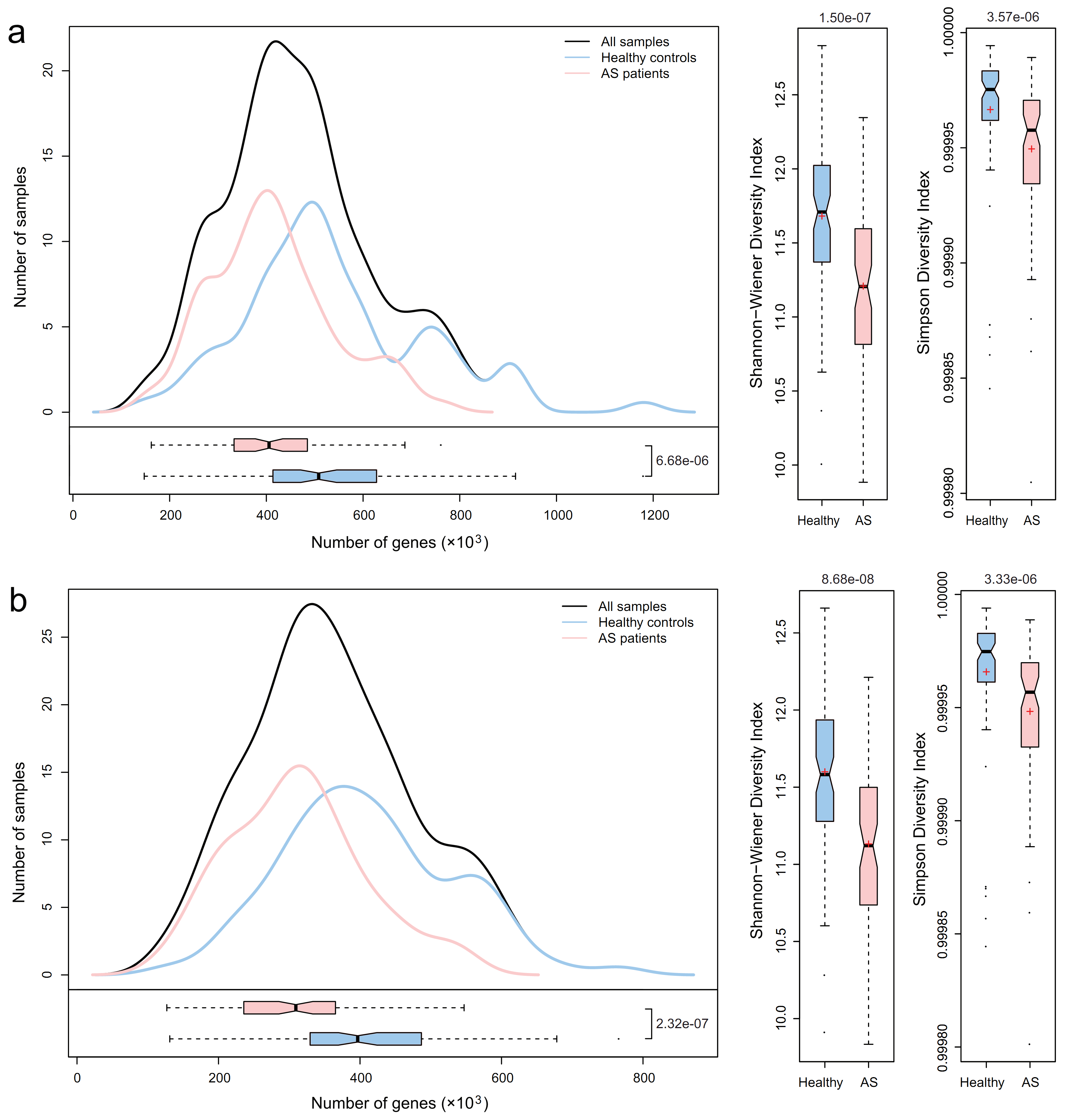


**Figure S5. The distribution of p values.**

The genes whose median abundances in two cohorts were both less than 1E-7 were filtered. After filtering, to identify the differentially abundant genes wilcoxon rank-sun test was applied. **a,** the distribution of p values is shown as histogram**,** histogram with ‘breaks=50,000’ was the details of the histogram with ‘breaks=500’, red line (FDR=0.001) was the cutoff of the differentially abundant genes. **b**, The distribution of p values in IBD project (red), LC project (green) and AS project (blue) is shown as polyline. .


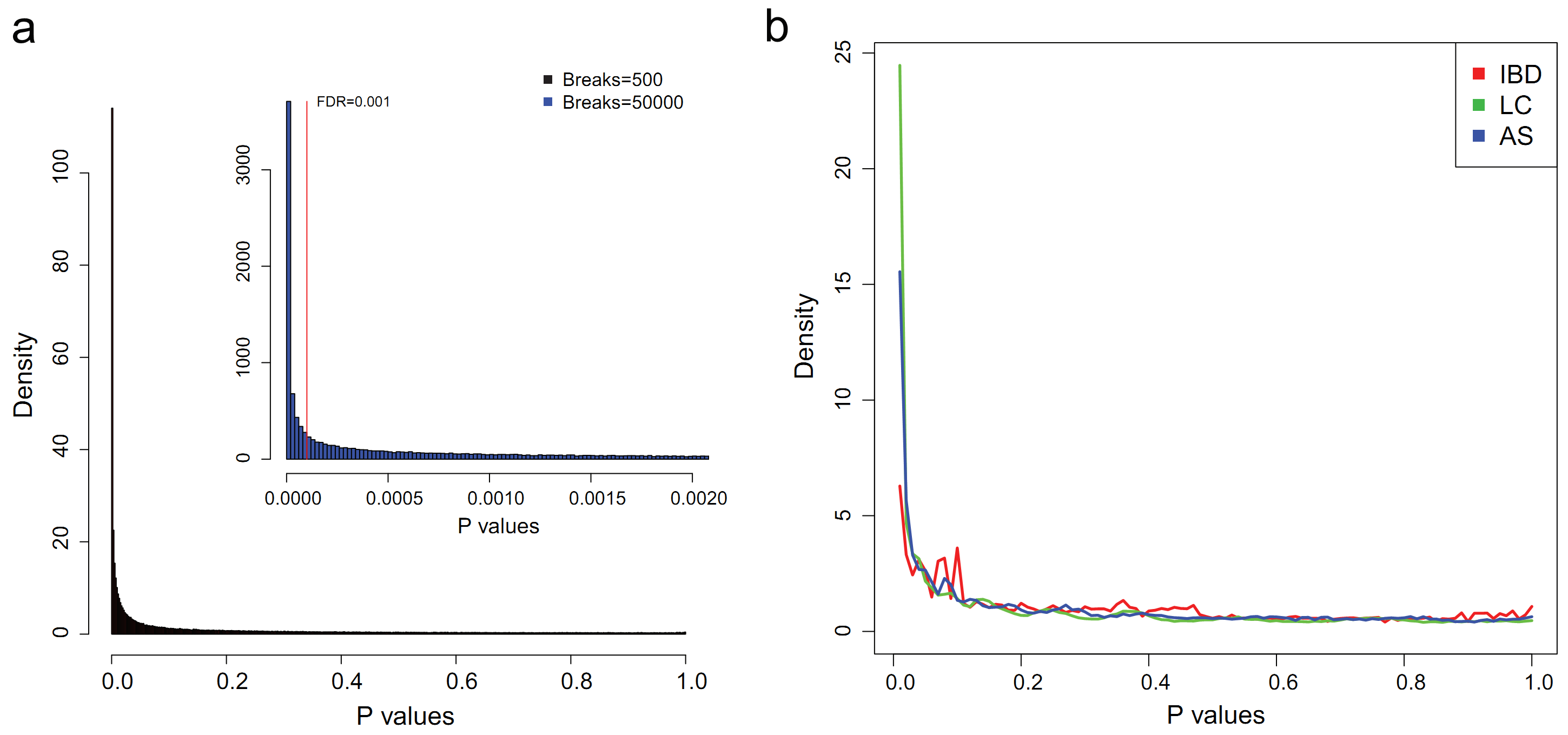


**Figure S6. The distribution of KEGG functional categories (statistics in Level 2) for all genes and differentially abundant genes.**

The distribution of KEGG functional categories for all identified genes shown by number (**a**) and percentage (**b**); the distribution of KEGG functional categories for AS-enriched genes and control-enriched genes shown by number (**c**) and percentage (**d**).


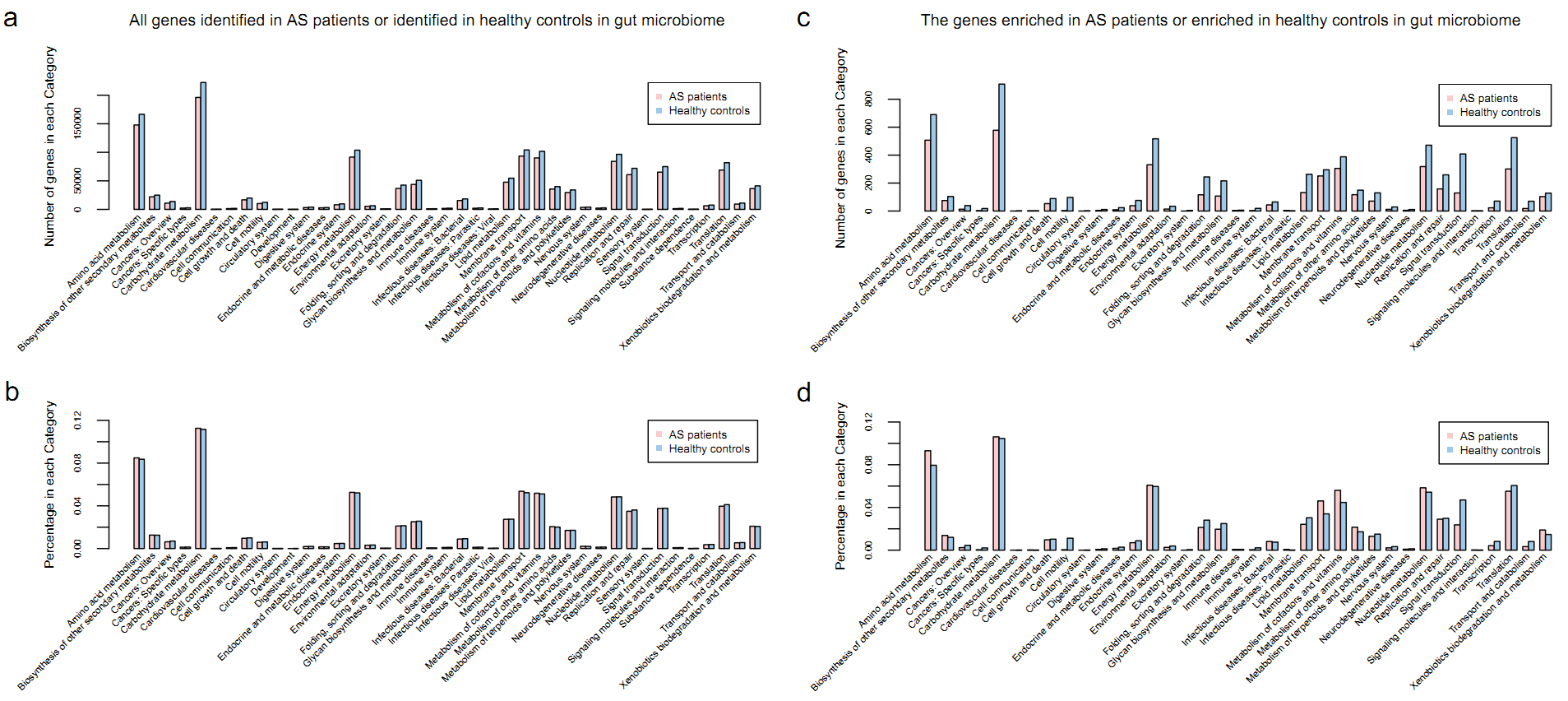


**Figure S7. The distribution of detail pathways in four KEGG functional categories which were quite different between AS-enriched genes and control-enriched genes in Figure S6.**

The distribution of detail pathways in four KEGG functional categories (Cell motility, Membrane transport, Metabolism of cofactors and vitamins, Signal transduction) for differentially abundant genes shown by number (**a**) and percentage (**b**).


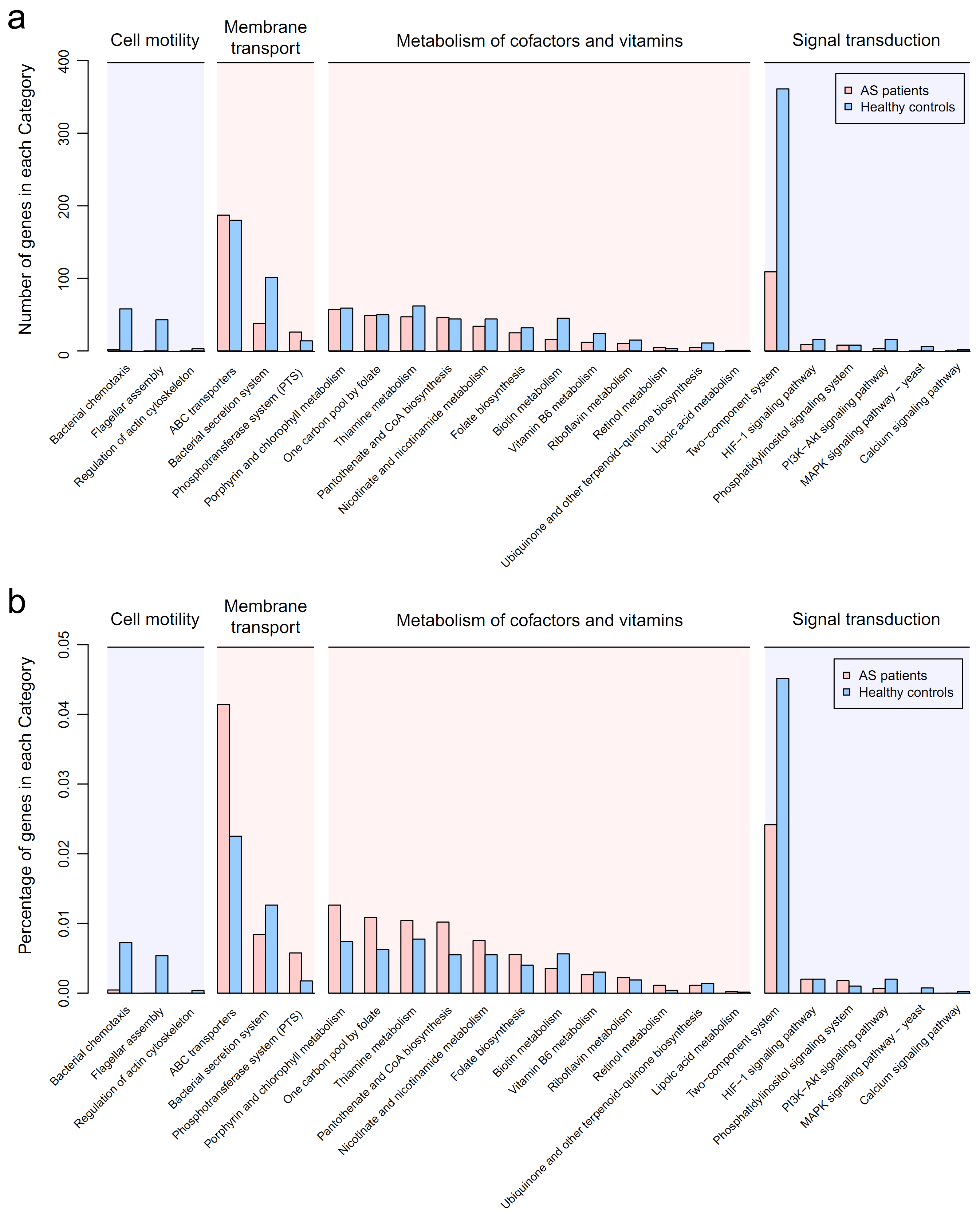


**Figure S8. The distribution of eggNOG functional categories for AS related markers.**

Comparison between the AS-enriched and control-enriched gene markers for different eggNOG functional categories shown by number (a) and percentage (b).


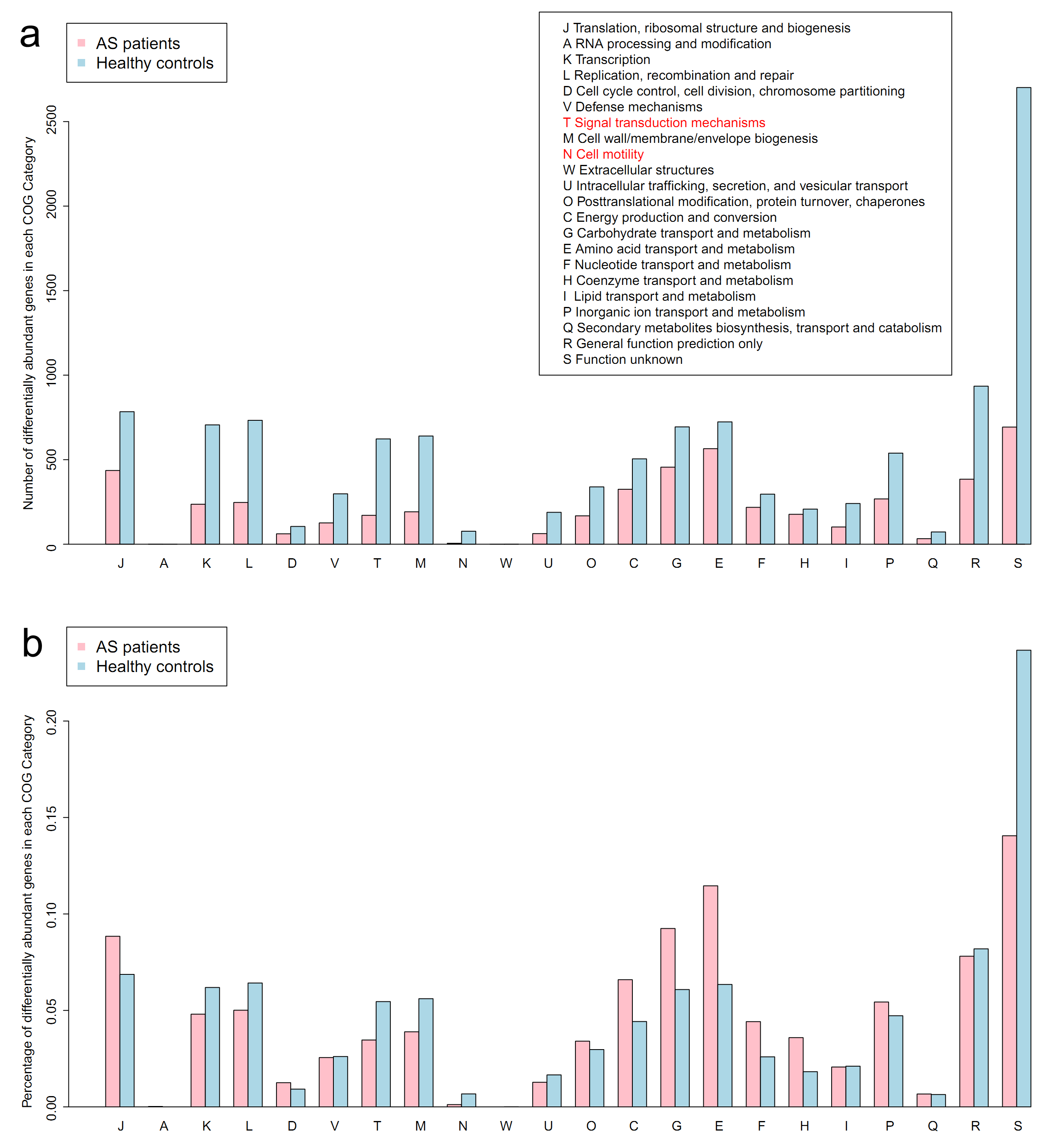


**Figure S9. The distribution of KEGG module categories for AS related markers shown by number and percentage.** AS-enriched gene markers were colored by red, control-enriched gene markers were colored by blue, other genes in each module were colored by green. The AS-enriched gene markers in Proteasome module were quite high by percentage.

**
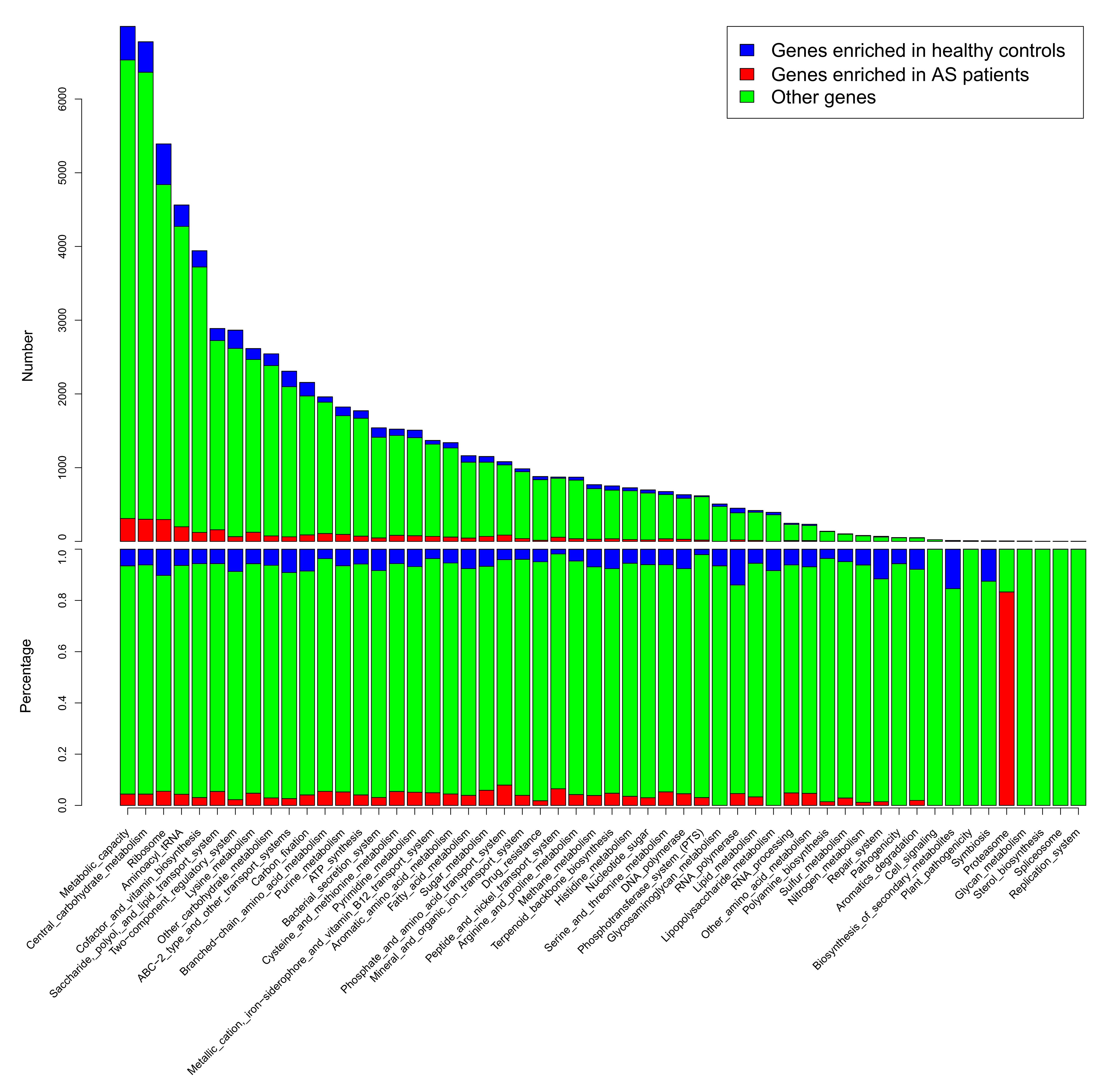
**

**Figure S10. Heatmap of the abundance of a random metagenomic species in both sequencing data and downloaded data.**

Genes in CAG1 are in rows of heatmaps: the left one is the abundance in AS patients and Healthy controls, while the right one is in other downloaded metagenomic data. Color varied by the abundance (white means highest, while black mean not detected). The color-keys on the top right corner showed the correlation between colors and abundance.


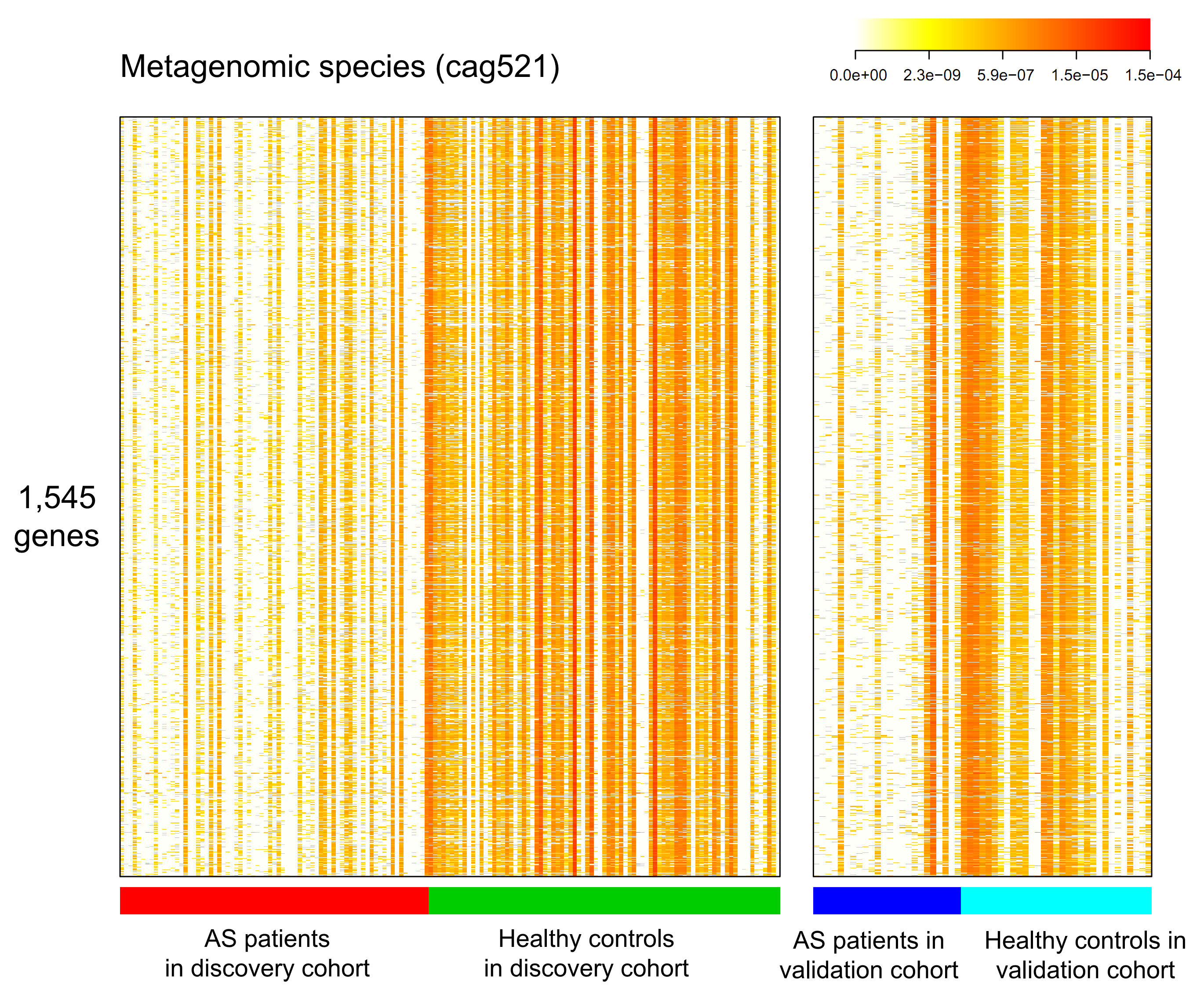


**Figure S11. Taxonomic annotation of genes in CAGs by NT database.**

All 199 CAGs were shown as barplot to indicate the percentage of genes which can map to NT database. Blue means genes in each CAG can map to the same one species in NT database; green means genes can map to NT database but not be only one species; red means genes cannot map to NT database well.


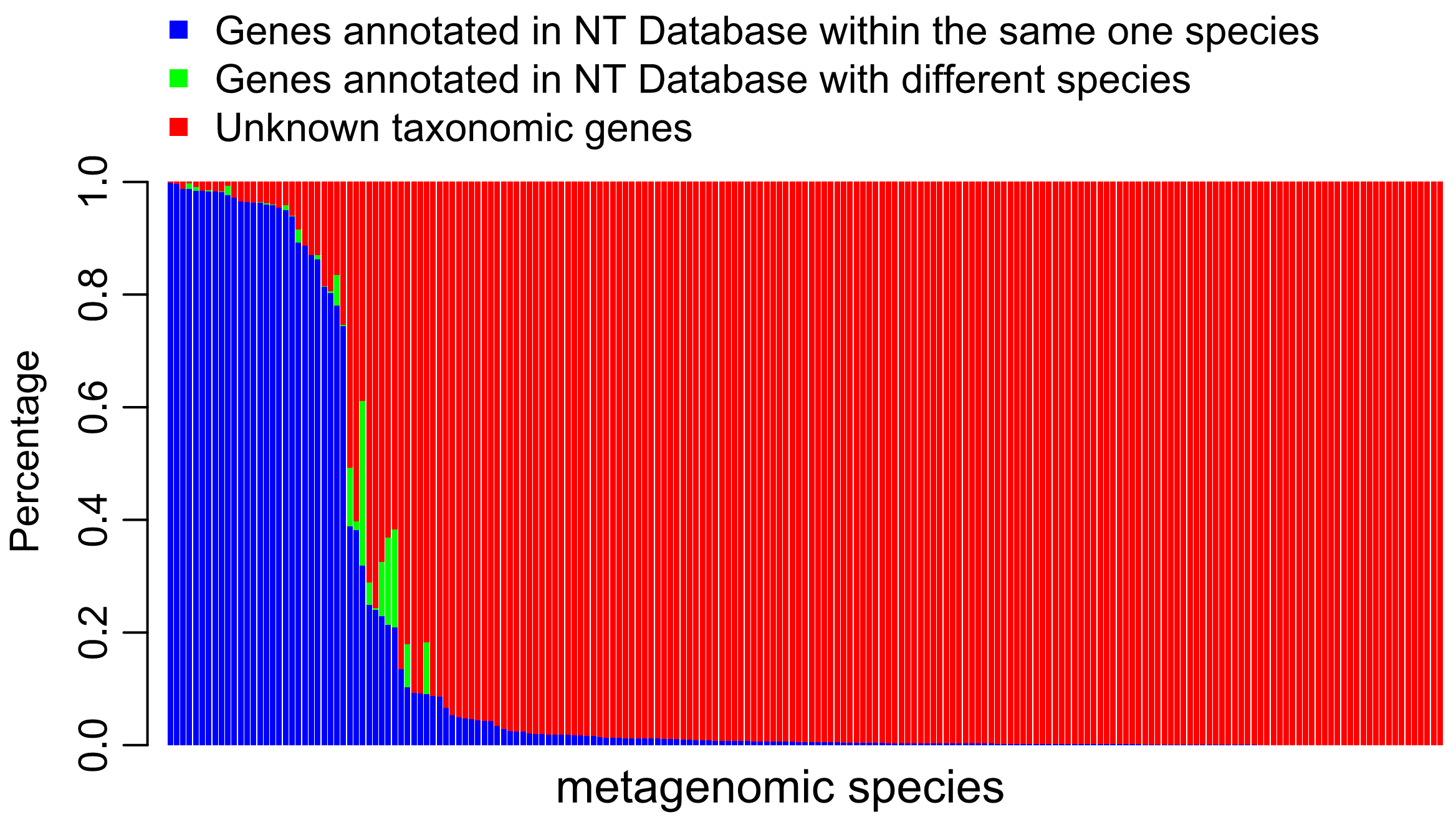


**Figure S12a. The NMDS (nonmetric multidimensional scaling) analysis based on phylogenetic abundance profiling of all the 156 samples in discovery cohort.** The color key shows the different ages. The barplots in the bottom and right are grouped into three groups according to different age ranges (age<=35 years old, 35<age<45 years old, age>=45 years old). It shows that there is no obvious separation in NMDS analysis in the three groups according to different age ranges.


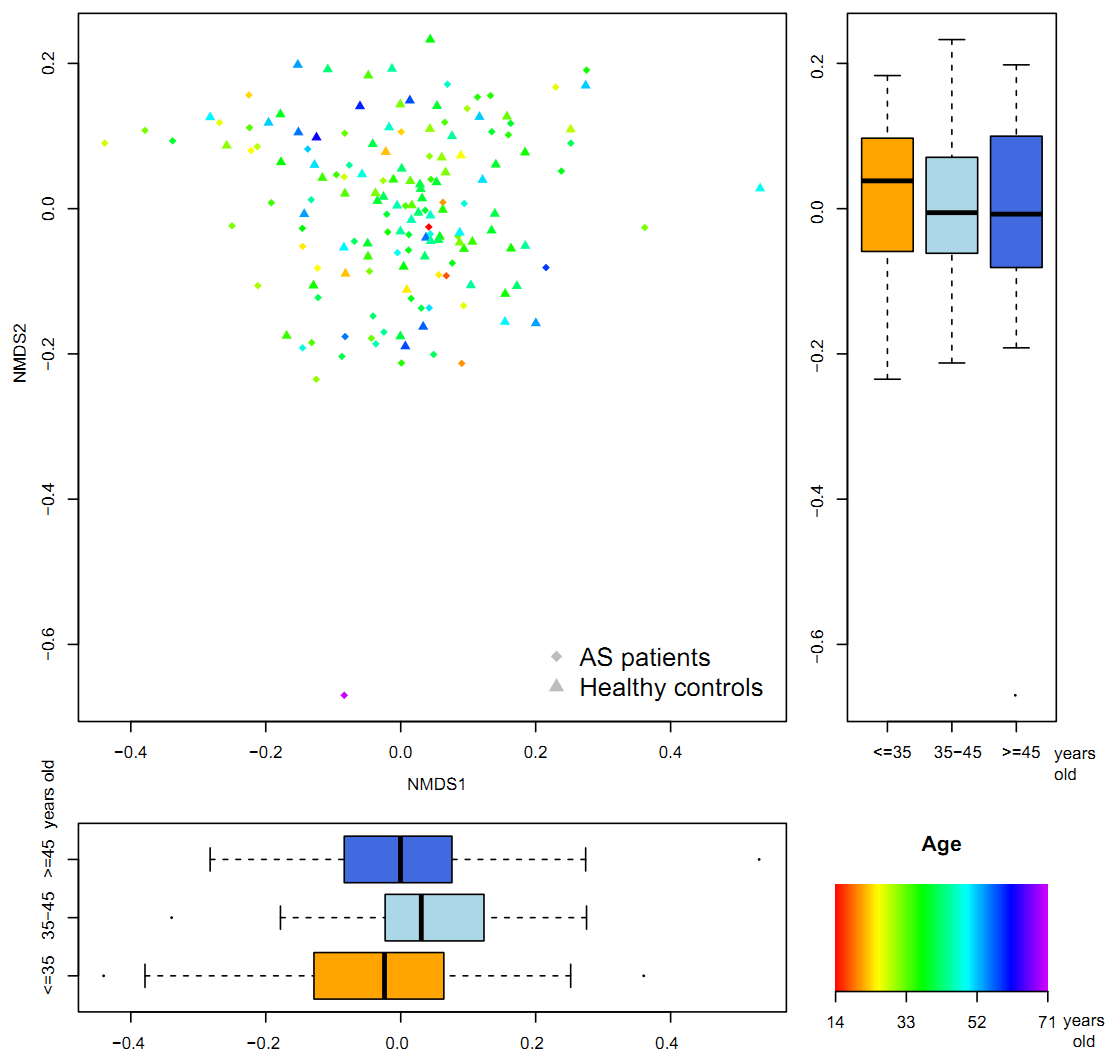


**Figure S12b. The NMDS (nonmetric multidimensional scaling) analysis based on phylogenetic abundance profiling of all the 156 samples in discovery cohort.** The color key shows the different BMI (Body Mass Index). The barplots in the bottom and right are grouped into three groups according to different BMI ranges (BMI<=20, 20<BMI<=24, BMI >24). It shows that there is no obvious separation in NMDS analysis in the three groups according to different BMI ranges.


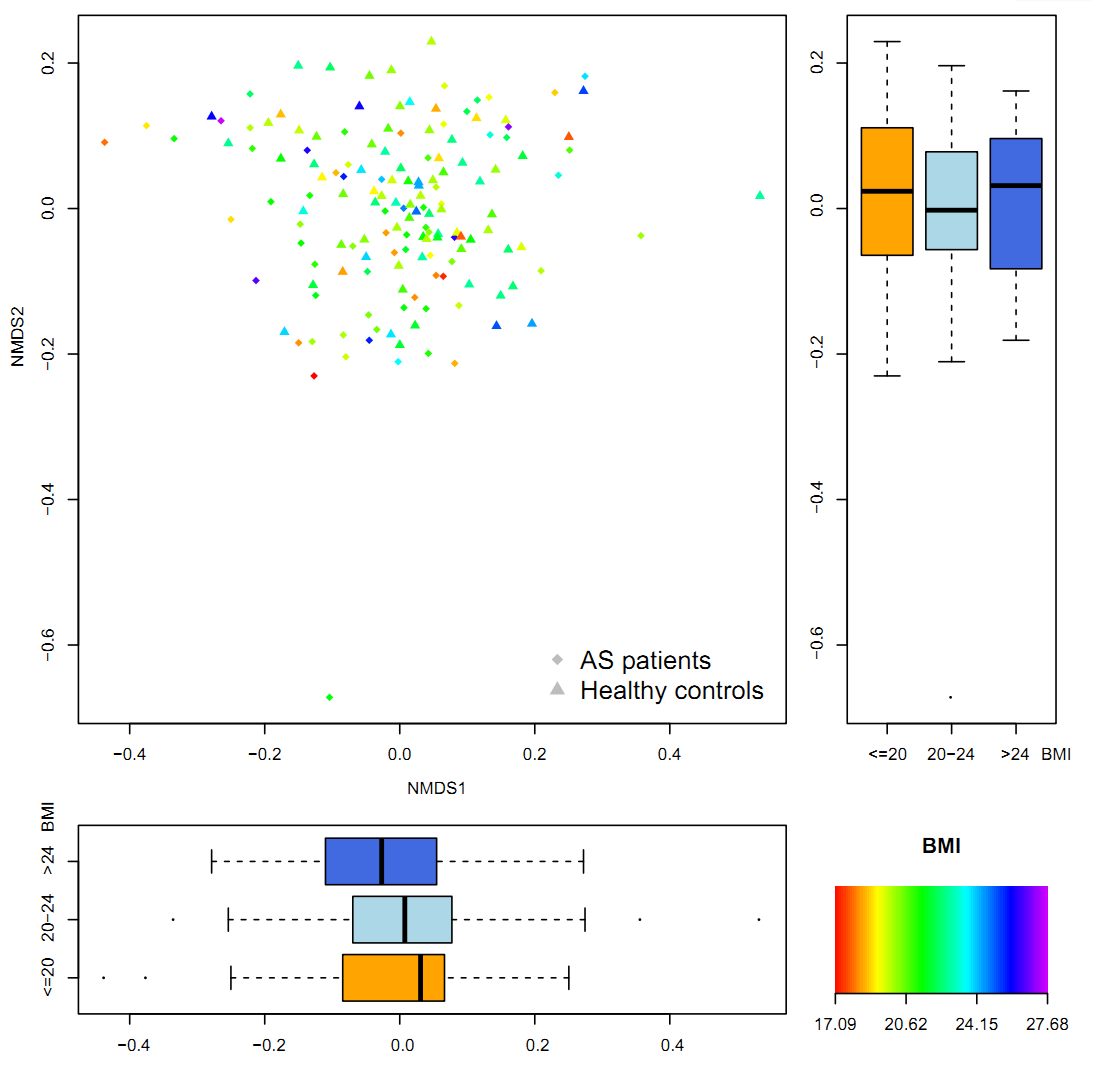


**Figure S12c. The NMDS (nonmetric multidimensional scaling) analysis based on phylogenetic abundance profiling of all the 73 AS samples in discovery cohort.** The different colors represent the different disease duration in AS patients. The barplots in the bottom and right are grouped into three groups according to different disease duration ranges (disease duration <=2 years, 2<disease duration<5 years, disease duration>=5 years). It shows that there is no obvious separation in NMDS analysis in the three groups according to different disease duration ranges.


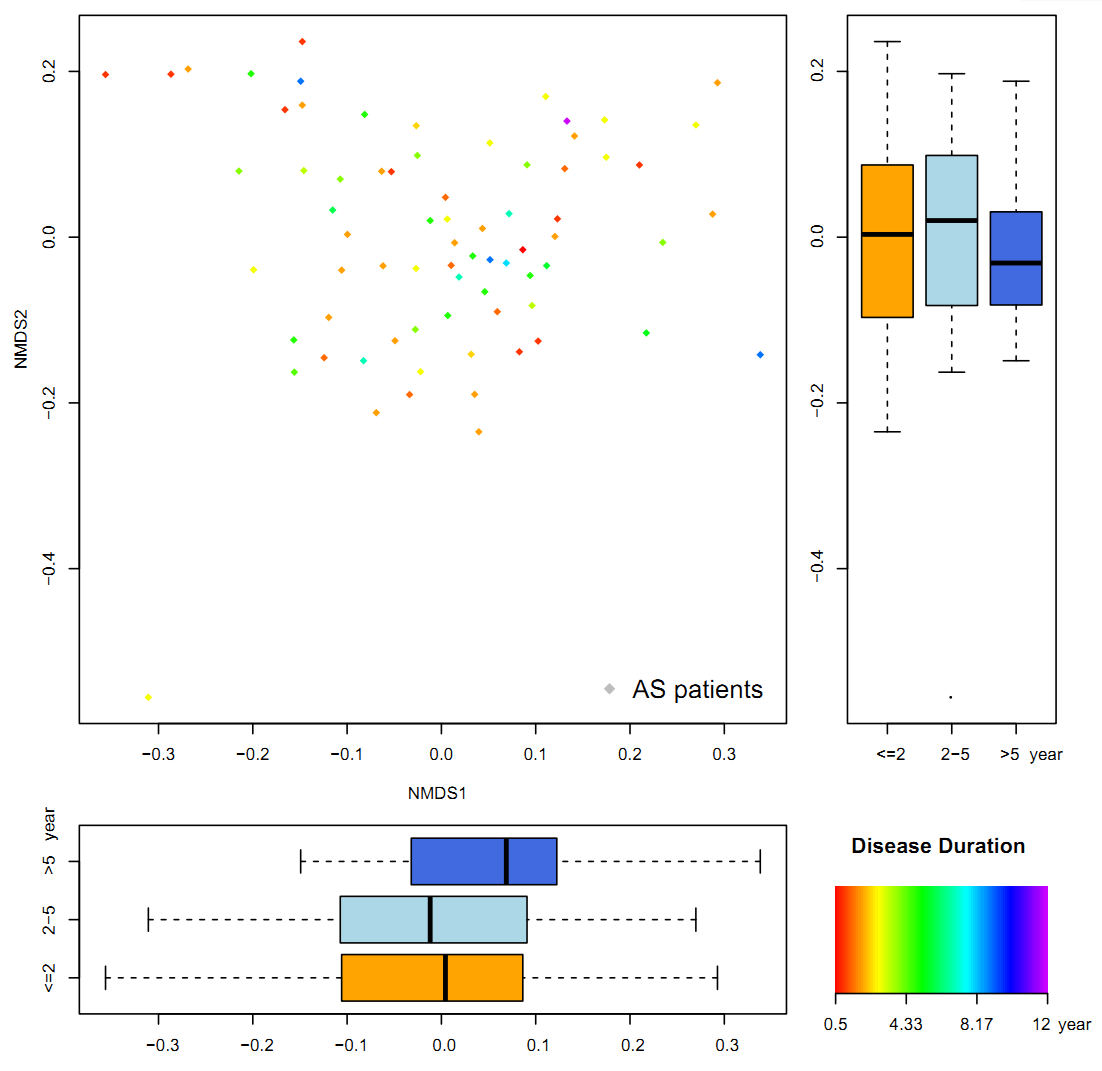


**Figure S12d. The NMDS (nonmetric multidimensional scaling) analysis based on phylogenetic abundance profiling of all the 73 AS samples in discovery cohort.** The blue points represent the AS patients who had treatment with NSAIDs currently, the red points represent the AS patients who did not have treatment with NSAIDs. The barplots in the bottom and right were grouped into these two groups. It shows that there is no obvious separation in NMDS analysis between the group with NSAIDs and the group without NSAIDs.


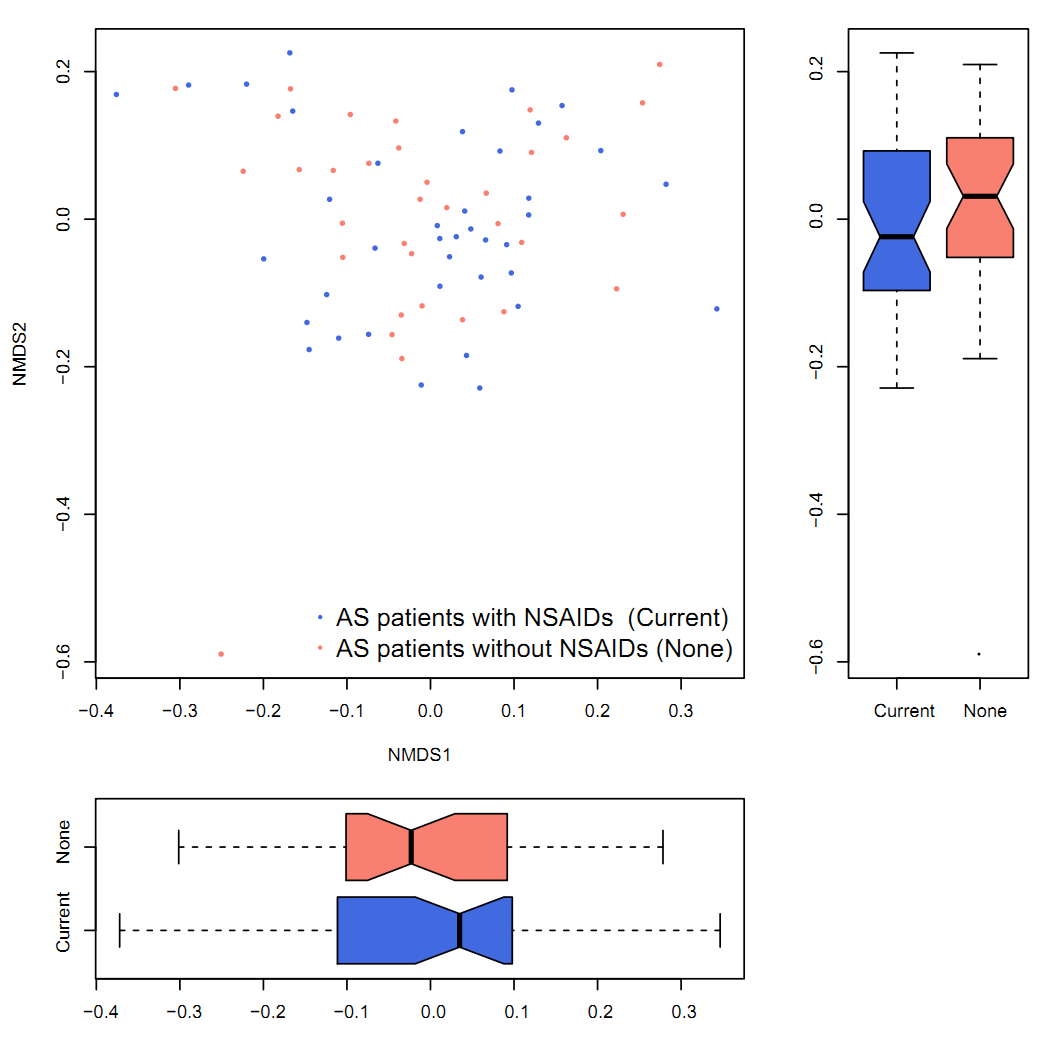

Supplement: Supplementary file 2 — Figure S1a. Venn diagram of three existing human gut gene catalogs. Figure S1b. Diversity of genera and species between AS patients and healthy controls. Figure S2. The Bacteroidetes/Firmicutes ratio in the AS patient group and in the healthy control group. Figure S3. Phylogenetic abundance under phylum, genus, and species levels between AS patients and healthy controls. Figure S4. Loss of richness of the gut microbiome in AS. Figure S5. The distribution of p values. Figure S6. The distribution of KEGG functional categories (statistics in Level 2) for all genes and differentially abundant genes. Figure S7. The distribution of detail pathways in four KEGG functional categories which were quite different between AS-enriched genes and control-enriched genes in Figure S6. Figure S8. The distribution of eggNOG functional categories for AS related markers. Figure S9. The distribution of KEGG module categories for AS related markers shown by number and percentage. Figure S10. Heatmap of the abundance of a random metagenomic species in both sequencing data and downloaded data. Figure S11. Taxonomic annotation of genes in CAGs by NT database. Figure S12. The NMDS (non-metric multidimensional scaling) analysis based on phylogenetic abundance profiling of all the 156 samples in the discovery cohort. (DOCX 4671 kb) [file 13059_2017_1271_MOESM2_ESM.docx]
